# Arabidopsis *TRANSCURVATA1* Encodes NUP58, a Component of the Nucleopore Central Channel

**DOI:** 10.1371/journal.pone.0067661

**Published:** 2013-06-28

**Authors:** Almudena Ferrández-Ayela, María Magdalena Alonso-Peral, Ana Belén Sánchez-García, Rosa Micol-Ponce, José Manuel Pérez-Pérez, José Luis Micol, María Rosa Ponce

**Affiliations:** Instituto de Bioingeniería, Universidad Miguel Hernández, Campus de Elche, Elche, Spain; Tulane University Health Sciences Center, United States of America

## Abstract

The selective trafficking of proteins and RNAs through the nuclear envelope regulates nuclear-cytoplasmic segregation of macromolecules and is mediated by nucleopore complexes (NPCs), which consist of about 400 nucleoporins (Nups) of about 30 types. Extensive studies of nucleoporin function in yeast and vertebrates showed that Nups function in nucleocytoplasmic trafficking and other processes. However, limited studies of plant Nups have identified only a few mutations, which cause pleiotropic phenotypes including reduced growth and early flowering. Here, we describe loss-of-function alleles of Arabidopsis *TRANSCURVATA1* (*TCU1*); these mutations cause increased hypocotyl and petiole length, reticulate and asymmetrically epinastic leaf laminae of reduced size, and early flowering. *TCU1* is transcribed in all of the organs and tissues examined, and encodes the putative ortholog of yeast and vertebrate Nup58, a nucleoporin of the Nup62 subcomplex. Nup58 forms the central channel of the NPC and acts directly in translocation of proteins through the nuclear envelope in yeast and vertebrates. Yeast two-hybrid (Y2H) assays identified physical interactions between TCU1/NUP58 and 34 proteins, including nucleoporins, SCF (Skp1/Cul1/F-box) ubiquitin ligase complex components and other nucleoplasm proteins. Genetic interactions were also found between *TCU1* and genes encoding nucleoporins, soluble nuclear transport receptors and components of the ubiquitin-proteasome and auxin signaling pathways. These genetic and physical interactions indicate that TCU1/NUP58 is a member of the Nup62 subcomplex of the Arabidopsis NPC. Our findings also suggest regulatory roles for TCU1/NUP58 beyond its function in nucleocytoplasmic trafficking, a hypothesis that is supported by the Y2H and genetic interactions that we observed.

## Introduction

In eukaryotes, the nuclear envelope spatially separates certain key cellular processes, segregating them to the nucleus or the cytoplasm. This segregation depends on controlled nucleocytoplasmic traffic mediated by nucleopore complexes (NPCs), which allow regulated transport of macromolecules and passive diffusion of ions and small molecules [1]. The NPCs of *Saccharomyces cerevisiae* and vertebrates include multiple copies of about 30 different nucleoporins (Nups). Nups have been classified into three categories: (a) transmembrane Nups, which bind the NPC to the nuclear envelope, (b) FG-Nups, which contain phenylalanine-glycine (FG) repeats and (c) structural Nups, connecting transmembrane Nups to FG-Nups [2], [3]. FG-Nups are assumed to play a pivotal role in nuclear transport, acting as a selective permeability barrier and mediating the translocation of soluble nuclear transport receptors. In addition to the NPC, two types of soluble nuclear transport receptors also participate in nucleocytoplasmic traffic: nuclear export factors, which are involved in the exit of mRNA from the nucleus, and β-karyopherin family proteins, including importins and exportins, which transport proteins containing nuclear localization signals (NLSs) or nuclear export signals (NESs), respectively [4]. Nuclear import or export of proteins largely depends on their interaction with soluble nuclear transport factors, which recognize and bind the NLS or NES to their cargoes. Upon direct or indirect binding of a protein to its soluble nuclear receptor, the receptor and cargo are translocated together across the central channel of the nucleopore [5].

Little experimental evidence is available on nucleoporin function in plants. Several Arabidopsis nucleoporins have been identified based on similarity to their putative orthologs in yeast and vertebrates. The Arabidopsis orthologs of Nup155, Nup98, TPR (translocated promoter region) and GP210 (nuclear pore glycoprotein of 210 kDa) nucleoporins were identified in this way [6]. Other Arabidopsis nucleoporins, including WIP1 (WPP-domain Interacting Protein1), WIP2, WIP3 and NUA (Nuclear Pore Anchor, the Arabidopsis ortholog of TPR) were first identified *in silico* in a search for proteins with a coiled-coil coverage of at least 50% and either a NLS or at least one predicted transmembrane domain [7]; the WIP proteins were then found to colocalize at the nuclear envelope [8].

Mutant screens have also led to the identification of nucleoporins in Arabidopsis. For several Arabidopsis genes encoding nucleoporins, loss-of-function mutations cause pleiotropic phenotypes that include reduced growth and early flowering. In vertebrates, the Nup107–160 nucleopore subcomplex comprises Nup96, Nup107, Nup133 and Nup160. The Arabidopsis genes encoding NUP96 and NUP160 have been identified in several mutant screens performed with diverse objectives. For example, the *mos3* (*modifier of snc1, 3*) and *mos7* mutants were isolated in a screen for suppressors of *snc1* (*suppressor of npr1-1*, *constitutive1*), a mutation that causes constitutive activation of disease resistance responses [9]. *MOS3* encodes NUP96 and *mos3-1* mutants exhibit small rosettes and early flowering. *MOS7* encodes NUP88, which is required for innate immunity and nuclear accumulation of defense regulators [10]. The Arabidopsis *sar1* (*suppressor of auxin resistance1*) and *sar3* mutants were identified in a search for suppressors of the auxin resistance phenotype of *axr1* (*auxin resistance1*). Additional *sar1* alleles were isolated in a search for mutations impairing the cold-induced transcription of the *CBF3-LUC* (*C-repeat/DRE Binding Factor 1-Luciferase*) reporter gene [11]. *SAR1* encodes NUP160, and *sar3* mutations are allelic to *mos3*. Single *sar1* and *sar3* and double *sar1 sar3* mutants display altered leaf morphology, early flowering, reduced rosette size and cell division rate in the primary root, and nuclear poly(A)^+^ RNA retention [12], [13].

In yeast, the TREX-2 complex (Transcription-coupled export 2) is anchored to the inner region of the NPC through Nup1, and is essential for mRNA export [14]-[18]. The TREX-2 complex includes the Thp1, Sac3, Sus1 and Cdc31 proteins. A yeast-two-hybrid (Y2H) screen using THP1 (Tho2/Hpr1 Phenotype) as bait identified a putative Arabidopsis ortholog of yeast Nup1, as well as other nucleoporins [19]. Mass-spectrometry analysis of proteins that coprecipitate with the RAE1 (RNA export factor 1) nucleoporin identified 30 components of the Arabidopsis NPC, of which 8 were previously described [20].

The central channel of the mammalian and yeast nucleopores is formed by the Nup54, Nup58 and Nup62 FG-nucleoporins of the Nup62 subcomplex, which in mammals also includes Nup45, a splice variant of Nup58. The Nup62 subcomplex is directly involved in the traffic of macromolecules across the nuclear envelope [21]. Here, we report the positional cloning of the *TRANSCURVATA1* (*TCU1*) gene of Arabidopsis, which encodes a Nup58 ortholog. The perinuclear localization of TCU1, the phenotypic effects of *tcu1* mutations, and the phenotypes of their double mutant combinations with alleles of genes involved in nucleocytoplasmic trafficking support that TCU1 is a nucleoporin. A Y2H-based screen of Arabidopsis cDNA libraries allowed us to identify TCU1 interactors including NUP62 and other nuclear proteins, some of which are components of the ubiquitin-proteasome and auxin signaling pathways.

## Materials and Methods

### Plant Materials and Growth Conditions

Seeds of the *Arabidopsis thaliana* L. Heynh. wild-type accessions L*er* and Col-0 were obtained from the Nottingham Arabidopsis Stock Centre (NASC). The *tcu1-1* mutant was isolated in the L*er* background after EMS mutagenesis [22]. Seeds of T-DNA insertion lines were provided by the NASC or ABRC (Table S1) and are described at SIGnAL (http://signal.salk.edu) [23]. Seed sterilization and sowing, plant culture and crosses were performed as previously described [22], [24]. Briefly, seeds were sown on plates containing MS agar medium (half-strength Murashige and Skoog salts, 0.7% plant agar [Duchefa], pH 5.7, and 1% sucrose) and stratified (4°C in the dark) for 48 h and then transferred to either Conviron TC16 or TC30 growth chambers set to standard conditions of continuous light at approximately 75 µmol·m^−2^·s^−1^, 20°C, 60–70% relative humidity. When required, plants were transferred into pots containing a 2∶2∶1 mixture of perlite:vermiculite:sphagnum moss and grown in walk-in growth chambers set to the same standard conditions.

### Plant Gross Morphology, Histology and Histochemical Assays

Leaf clearing and fixation, embedding, microscopy and morphometry were performed as previously described [25]–[28]. Venation, leaf epidermal and mesophyll cell diagrams were obtained from micrographs by hand drawing on the screen of a Wacom Cintiq 18SX Interactive pen display (http://www.wacom.com/) and using the Adobe Photoshop CS3 (http://www.adobe.com) software. Morphometric analyses of the diagrams (n≥10) were performed with ImageJ 1.36b [29] (http://rsb.info.nih.gov/ij/index.html/), Scion Image 4.0.3.2 and NIS-Elements AR 2.30 (Nikon Imaging; http://www.nis-elements.com/). GUS assays were performed as described in [30]. Unless otherwise indicated, all values in this paper are reported as means ± standard deviation from at least 10 (for morphometry) or 20 (all other experiments) plants.

### Positional Cloning and Molecular Characterization of *TCU1* and its Mutant Alleles

Low-resolution mapping of the *tcu1-1* mutation was performed as previously described [31], [32]. For the fine mapping of the *TCU1* gene, SSLP, SNP and In/Del markers were developed based on the polymorphisms between L*er* and Col-0 described in the Monsanto Arabidopsis Polymorphism Collection database (http://www.arabidopsis.org). Synthetic oligonucleotides were purchased from Sigma-Aldrich UK (Table S2). Genomic DNA was extracted, PCR amplified and sequenced as described previously [33]. For the sequencing of *tcu1* alleles, PCR amplification products spanning the At4g37130 transcription unit were obtained using wild-type and mutant genomic DNA as templates. To confirm the presence and position of T-DNA inserts, DNA was extracted and PCR amplified. Sequencing reactions, RNA extractions and qRT-PCR amplifications were performed as described in Barrero et al. [34], using the primers shown in Table S3. For qRT-PCR, each reaction was made using three biological replicates, each with three technical replicates; the expression levels were normalized to the C_T_ values obtained for the housekeeping gene *OTC* [35].

### DNA Constructs

To complement the *tcu1-1* mutation, a 3.2-kb L*er* genomic fragment containing the *TCU1* coding region and its promoter was PCR amplified using the oligonucleotides indicated in Table S3. The amplification product was cloned into the *EcoR*I and *Xba*I sites of pGreen0179 (http://www.pgreen.ac.uk/JIT/pG0179.htm). The resulting *TCU1_pro_:TCU1* construct was mobilized into *Agrobacterium tumefaciens* C58C1-pSOUP cells and then transferred into Col-0, L*er* and *tcu1-1* plants.

Gateway (Invitrogen) entry and destination vectors were used to obtain all the remaining constructs used in this work using the oligonucleotide primers indicated in Table S3. We constructed the *TCU1_pro_:GUS* transgene to visualize the expression pattern of the *TCU1* gene, *TCU1_pro_:TCU1:GFP* to visualize the subcellular localization of the TCU1 protein, and *2x35S:TCU1* and *2x35S:tcu1-1* to overexpress the *TCU1* and *tcu1-1* alleles. We PCR amplified genomic fragments containing the *TCU1* promoter (443 bp from L*er*) for *TCU1_pro_:GUS*; the *TCU1* promoter and coding region, lacking its stop codon (2,7 kb from L*er*) for *TCU1_pro_:TCU1:GFP*; and the whole *TCU1* coding region (2,3 kb from either L*er* or *tcu1-1*) for *2x35S:TCU1* and *2x35S:tcu1-1*, respectively. The amplification products were cloned into the pGEM-T Easy221 vector, sequence verified, transferred into the pMDC164, pMDC111 and pMDC32 destination vectors, respectively, and sequence verified again. The resulting constructs were mobilized into *Agrobacterium tumefaciens* LBA4404 cells, and then transferred into Col-0, L*er*, *tcu1-1* and *tcu1-2* plants. Arabidopsis plant transformations were performed by the floral dip method [36], and the transgenic plants were selected on MS agar medium supplemented with 15 µg·ml^−1^ hygromicin.

### Ubiquitin Immunodetection

Protein extraction and immunoblotting were performed as described in [37]. Antibody (antiUBQ11; Agrisera) serum [38] was used at a dilution of 1∶1,000 (v/v). The immunoreactive proteins were visualized using Pierce picosignal reagents, with the secondary rabbit antibody diluted 1∶5,000 (v/v), and by exposure to X-ray film (Amersham Hyperfilm) for 10 s to 1 h.

## Results

### The *tcu1-1* Mutation Causes a Pleiotropic Phenotype

We performed a large-scale screen for EMS-induced mutants with altered leaf morphology in the L*er* genetic background. Some mutants exhibited leaves folded towards the abaxial surface in an asymmetrical manner relative to the primary vein. We called these mutants *tcu* (*transcurvata*). The *tcu* mutations are recessive and fully penetrant, with only small variations in expressivity, and fall into three complementation groups (*TCU1*, *TCU2* and *TCU3*) [22].

**Figure 1:**
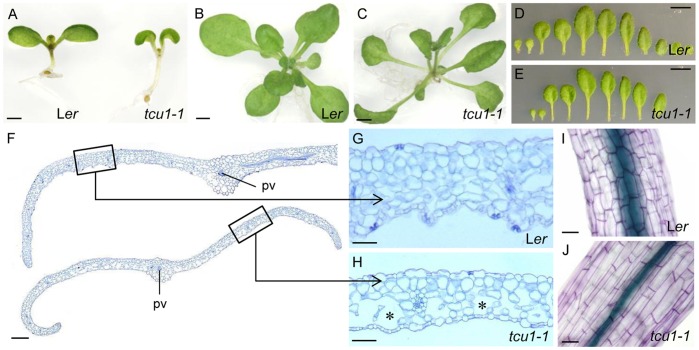
Morphological and cellular phenotypes of the *tcu1-1* mutant. (A) Lateral view of seedlings and (B, C) top view of rosettes of the indicated genotypes. (D, E) From left to right, dissected cotyledons and first to ninth (in L*er*) or seventh (in *tcu1-1*) vegetative leaves. (F-H) Leaf transverse sections and (I, J) hypocotyl cells from plants of the indicated genotypes. pv: primary vein. Asterisks in H highlight enlarged intercellular air spaces. Plants were collected at (A) 12, (B, C, F–J) 21 and (D, E) 22 das. Scale bars: (A) 1 mm, (B, C) 2 mm, (D, E) 5 mm, (F) 200 µm and (G–J) 50 µm.

The cotyledons of the *tcu1-1* mutant failed to expand completely; also, vegetative leaf laminae were slightly smaller than wild type (Figure 1A-E), and reticulated, with veins greener than the interveinal regions. These visible traits correlated with perturbations in internal leaf anatomy, as shown by the enlarged air spaces and fewer cells in the interveinal spongy mesophyll seen in transverse sections (Figure 1F-H). The leaf veins and their perivascular bundle sheath cells seemed unaffected. Differential interference contrast (DIC) microscopy of first- and third-node *tcu1-1* and L*er* leaves cleared with chloral hydrate revealed only small differences in cell number or cell size in the abaxial and adaxial epidermal layers, as well as in the subepidermal layer of palisade mesophyll cells (Figure S1). Similar to vegetative leaves, cauline leaves were folded downwards.

Hypocotyls of plants grown in our standard conditions were longer in *tcu1-1* (2.09±0.26 mm) than in L*er* (1.46±0.27 mm). Hypocotyl epidermal cell length was 194.85±32.79 µm in the mutant and 91.30±19.08 µm in the wild type (Figure 1I, J). The petiole and its adaxial epidermal cells were longer in the *tcu1-1* mutant (4.42±0.74 mm and 444.94±168.14 µm, respectively) than in L*er* (3.09±0.46 mm and 320.41±134.40 µm) in first-node leaves collected 13 das (days after stratification). Bolting was observed at 21.86±0.94 das in *tcu1-1* and 27.89±1.36 das in L*er*, and the mutant developed fewer vegetative leaves (7.36±0.81) than L*er* (12.40±0.52).

No differences with the wild type were observed in root, inflorescence, flower and fruit morphology, root gravitropism and skotomorphogenesis. The venation patterns of *tcu1-1* leaves had slightly increased total vein length and number of free-ending veins, but reduced number of branching points compared with L*er* (Figure S2 and Table S4).

### The *TCU1* Gene Encodes the Arabidopsis NUP58 Nucleoporin

**Figure 2:**
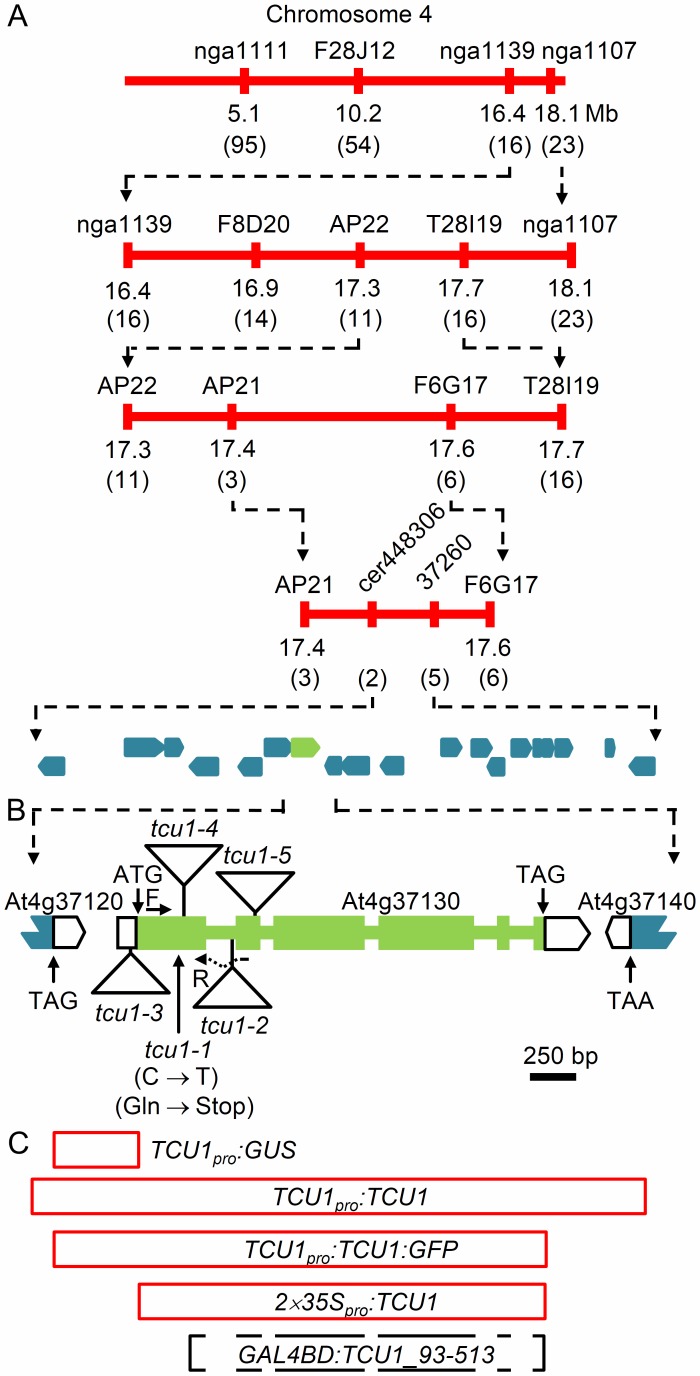
Positional cloning, structure and constructs of the *TCU1* gene. (A) Map-based cloning strategy. The molecular markers used for linkage analysis and the number of informative recombinants identified (in parentheses) are indicated. (B) Schematic representation of the *TCU1* gene and *tcu1* mutant alleles. Triangles represent T-DNA insertions. Exons are shown as boxes, and introns as lines between boxes. Open boxes indicate 5′ and 3′ untranslated regions. Translation start and stop codon positions are shown. Horizontal arrows represent oligonucleotides used as primers for qRT-PCR amplifications (not drawn to scale). (C) Span of the genomic DNA (red) or cDNA (black) segments amplified by PCR to obtain the constructs shown, which were used for the functional characterization of *TCU1*.

For the positional cloning of *TCU1*, we performed linkage analysis on an F_2_ mapping population of 740 plants derived from a *tcu1-1*× Col-0 cross (see Materials and Methods). This allowed us to delimit a 77-kb candidate region on chromosome 4, encompassing 19 annotated genes (At4g37070-At4g37260) (Figure 2A). Sequencing of these genes in the *tcu1-1* mutant revealed a C→T transition predicted to create a premature stop codon at nucleotide position 184 (numbering from the predicted translation initiation codon) in At4g37130 (Figure 2B).

To confirm that loss of At4g37130 function causes the *tcu1-1* mutant phenotype, we conducted several transgene-mediated complementation experiments. A wild-type (L*er*) genomic segment encompassing the entire region between At4g37120 and At4g37140 in the *TCU1_pro_:TCU1* transgene (Figure 2C) was transferred into *tcu1-1* plants, and all the resulting transformants displayed wild-type morphology (Figure S3). Another transgene including the full coding region of At4g37130 expressed under the control of the 35S promoter (*2x35S_pro_:TCU1*; Figure 2C) also rescued the phenotype of the *tcu1-1* mutant but had no phenotypic effects in the L*er* background. A construct carrying the same genomic segment of the mutant allele (*2x35S_pro_:tcu1-1*) did not cause any phenotypic effects in the L*er* or *tcu1-1* backgrounds.

**Figure 3:**
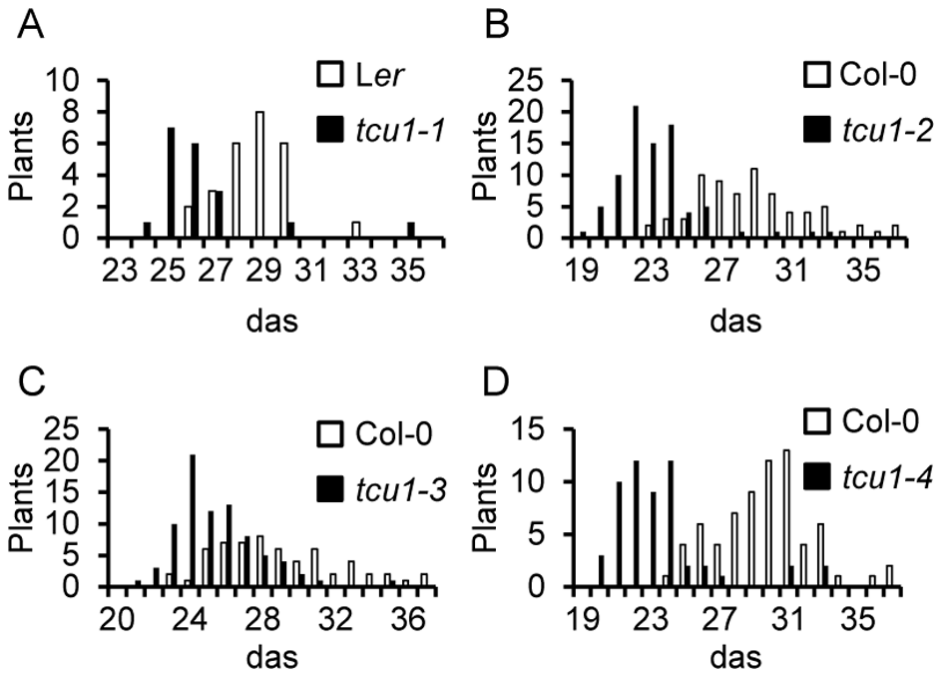
Early flowering in the *tcu1* mutants. Bolting time of (A) *tcu1-1* versus its wild type L*er*, and (B–D) *tcu1-2*, *tcu1-3* and *tcu1-4* versus their wild type Col-0.

Four publicly available lines carrying T-DNA insertions within the At4g37130 gene were identified (Table S1). Their insertions were sequence-confirmed to be at the annotated nucleotide positions. Allelism tests further confirmed that they carry *TCU1* alleles, which we called *tcu1-2* to *tcu1-5.* All these insertional lines shared phenotypic traits with *tcu1-1* to different degrees, and these shared phenotypes include early flowering (Figure 3) and increased petiole length. Leaf lamina folding and reduced size were similar in *tcu1-1* and *tcu1-4* mutants, but milder in the remaining lines.

**Figure 4:**
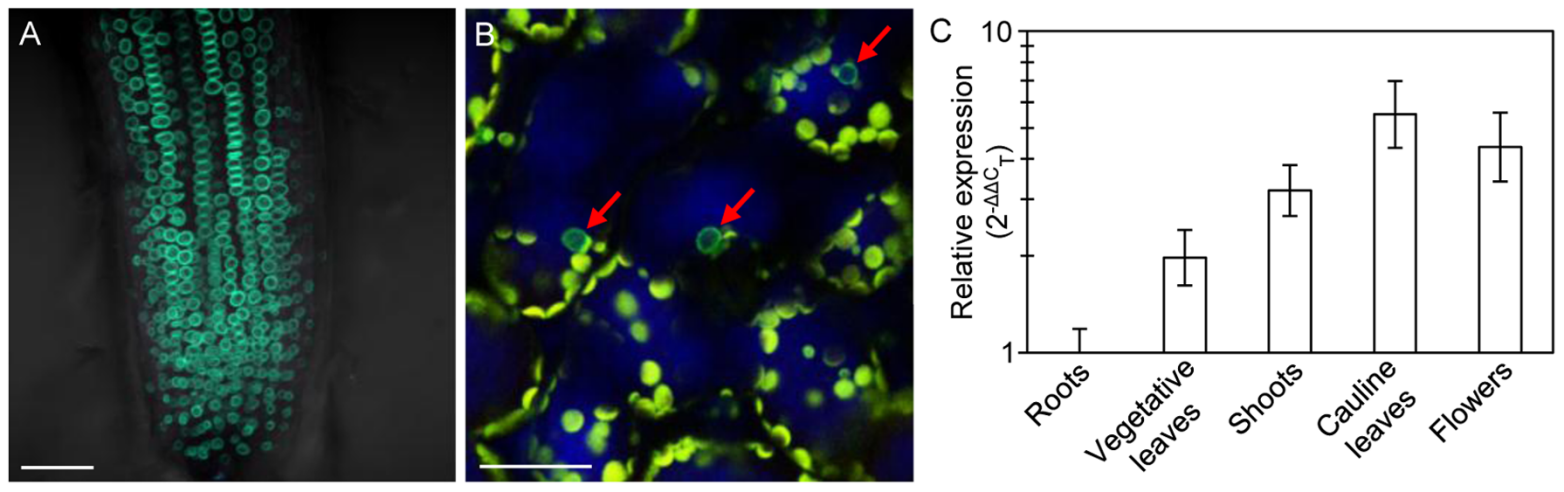
Subcellular localization of the TCU1:GFP protein and spatial pattern of expression of *TCU1*. (A, B) Confocal images of cells from (A) the root elongation zone and (B) the subepidermal layer of leaf palisade mesophyll from L*er* plants carrying the *TCU1_pro_:GFP* transgene. The green, perinuclear signal corresponds to GFP, and the yellow signal to chloroplast chlorophyll autofluorescence. Red arrows in B indicate nuclei. Scale bars: 20 µm. (C) qRT-PCR analysis of expression of *TCU1*. Total RNA isolated from the tissues indicated was used as a template. Error bars represent standard deviations.

*TCU1* is predicted to encode an FG-nucleoporin of 513 amino acids and a molecular mass of 56.5 kDa (http://www.arabidopsis.org/servlets/TairObject? id = 126619&type = locus), a putative member of a hydroxyproline-rich glycoprotein family, closely related to mammalian Nup58/45 (Figure S4). To further validate its identity as a nucleoporin, we determined the subcellular localization of TCU1 using a translational fusion to the Green Fluorescent Protein (GFP) marker, under the control of the native *TCU1* promoter, *TCU1_pro_:TCU1:GFP* (Figure 2C). This transgene completely complemented the morphological phenotype of *tcu1-1*, indicating that the GFP fusion did not impair TCU1 function. As expected for a predicted nucleoporin, the GFP signal was detected only along the nuclear envelope in root and leaf mesophyll cells (Figure 4).

### *TCU1* is Broadly Expressed

We examined in silico the expression profile of *TCU1* using Genevestigator (http://www.genevestigator.ethz.ch) [39] and the Arabidopsis eFP Browser (http://bbc.botany.utoronto.ca/efp/cgi-bin/efpWeb.cgi) and found that *TCU1* is expressed in all tissues and developmental stages in Arabidopsis (Figure S5). To confirm this expression pattern, we also performed real-time qRT-PCR amplifications using RNA from assorted tissues of Col-0 and L*er*. The highest and lowest expression levels of *TCU1* were detected in cauline leaves and roots, respectively (Figure 4C).

*TCU1* expression was further analyzed by transferring a *TCU1_pro_:GUS* construct (Figure 2C) into Col-0, L*er*, *tcu1-1* and *tcu1-2* plants, with similar results in all genetic backgrounds. GUS activity was detected in all the stages and tissues analyzed of 4, 7, 11, 15, 18 and 21 das transgenic plants (Figure S6), being most intense in leaf primordia and the vasculature. The highest expression levels were detected between 7 and 15 das.

### TCU1 Physically Interacts with Nucleoporins and Other Nuclear Proteins, Including ASK1 and ASK2

To gain insight into the function of *TCU1*, the Y2H system was used to screen for TCU1 interactors. Two Arabidopsis cDNA libraries, totalizing 21 million prey clones, were screened at PanBioNet (http://www.panbionet.com) using the TCU1_93-513 bait, which encompasses amino acids 93 to 513 of TCU1. This screen identified ninety prey clones, representing 34 different genes, 17 of which encode known nuclear proteins (Table S5 and Figure S7).

**Figure 5:**
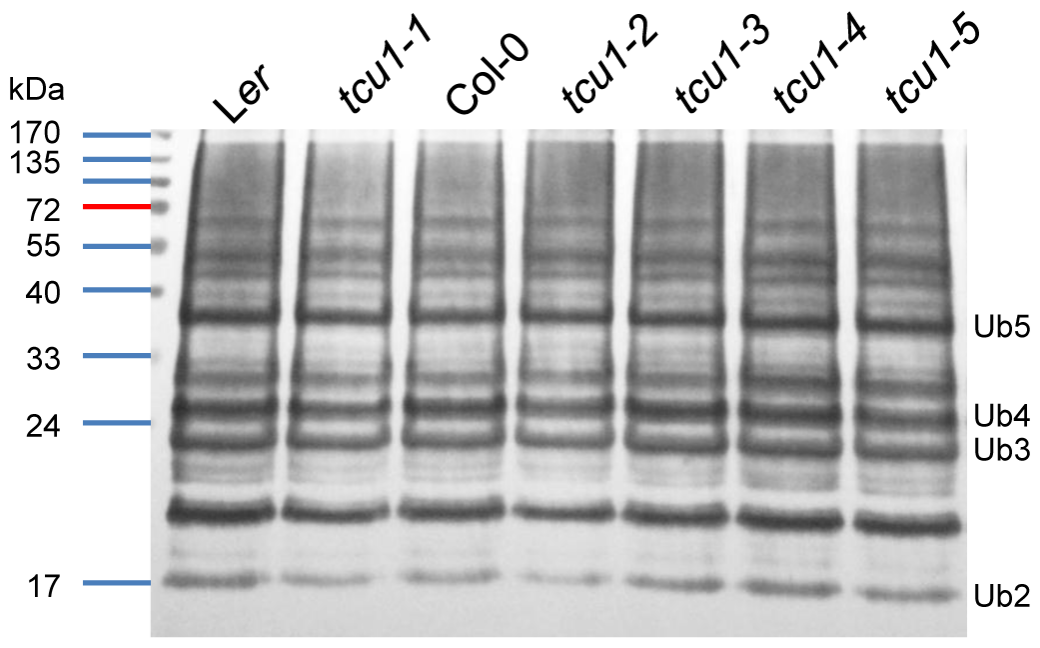
Ubiquitination in the *tcu1* mutants. Twenty micrograms of protein was electrophoresed on a 5% stacking/10% separating polyacrylamide gel, electroblotted onto nitrocellulose membrane and probed with an anti-ubiquitin (antiUBQ11) antibody. The positions of molecular mass markers are shown on the left, and those of some poly-ubiquitin chains on the right.

Proteins particularly well-represented in the Y2H-based screen included GAI (GA INSENSITIVE, 5 clones), which is a member of the DELLA family of growth repressors [40]-[42] and key negative regulators of gibberellin signaling [43]. Other identified proteins included NUP62 (also named EMB2766, 4 clones); ACT7 (4 clones), a component of the actin pathway [44]; the AT1G21440 mutase (4 clones); and the AT3G20720 unknown protein (4 clones). In most other cases only one or two positive clones were identified for a given protein. However, the screen also identified 8 ASK1 and 15 ASK2 prey clones. SKP1 (S PHASE KINASE-ASSOCIATED PROTEIN1), also called ASK1 (ARABIDOPSIS SKP1-LIKE1), and ASK2 are components of the SKP1-cullin/CDC53-F-box protein (SCF) ubiquitin-protein ligase complex, which plays important roles in selecting substrates for proteolysis by facilitating the ligation of ubiquitin to specific proteins [45], [46]. This interaction of TCU1/NUP58 with two SCF components prompted us to determine the levels of ubiquitin conjugates in the *tcu1* mutants. However, we found that the levels of high molecular mass polyubiquitinated proteins were not apparently altered in the *tcu1* mutants compared to their wild types (Figure 5).

### *TCU1* Genetically Interacts with Genes Required for Nucleocytoplasmic Trafficking and the Ubiquitin-proteasome Pathway

To examine the genetic interactions among genes encoding proteins involved in nucleocytoplasmic transport, we generated double mutants between *tcu1-1* and *tcu1-2* and loss-of-function mutations in genes encoding putative or known nucleoporins, soluble nuclear receptors and some of the interactors found in the Y2H screen (Table S5 and Figure S7).

**Figure 6:**
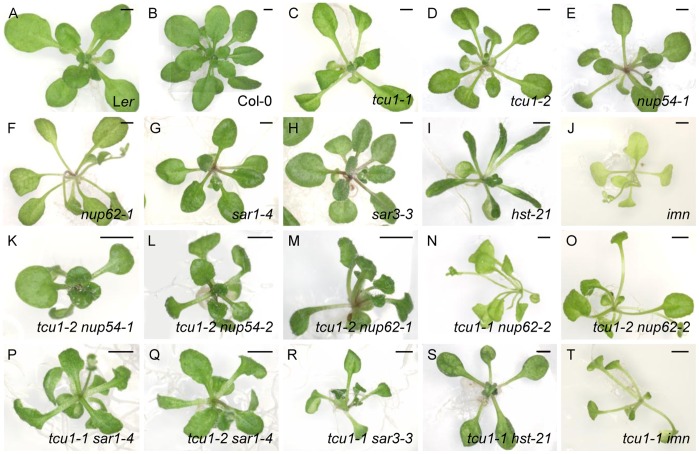
Genetic interactions between *tcu1* alleles and alleles of genes known or suspected to be involved in nucleocytoplasmic trafficking. All plants are homozygous for the mutations indicated. Pictures were taken at (A, B, I-T) 21, (C, D) 22, (E, F) 25 and (G, H) 18 das. Scale bars: 2 mm.

The *nup54-1* and *nup62-1* mutations (Figure 6E, F), and *nup54-2* and *nup62-2* to a lesser extent, and also caused early flowering, increased petiole length and reduced lamina size in vegetative leaves. A synergistic phenotype was seen in all double mutant combinations of *tcu1* alleles and *nup54-1*, *nup54-2* or *nup62-1* (Figure 6K-M), consisting of much smaller and darker rosettes than in their single mutant parental lines. A functional relationship between TCU1/NUP58, NUP54 and NUP62 was expected because they all belong to the Nup62 subcomplex. The *tcu1-1 nup62-2* and *tcu1-2 nup62-2* double mutants were pale and exhibited very long petioles (Figure 6N, O), phenotypes that were also considered to be synergistic.

We also observed synergistic phenotypes in the *tcu1 sar1* and *tcu1 sar3* double mutants (Figure 6P-R), an observation that indicates a functional relationship between *TCU1* and genes encoding components of the Nup160 nucleopore subcomplex. Mutations in genes encoding soluble nuclear receptors such as *hst-21* and *imn* interacted synergistically with *tcu1-1* and *tcu1-2*. HST (HASTY) is the Arabidopsis ortholog of mammalian exportin5 and yeast MSN5, two importin β-like soluble transport receptors. The HST protein is thought to regulate the nucleocytoplasmic transport of microRNA molecules [47]. The phenotype of the *hst-21 tcu1-1* double mutant differed from that of its single mutant parentals, with small rosettes, strongly reticulated leaves, short stems, flowers and fruits, and reduced fertility (Figure 6S). The strong hyponasty of *hst-21* leaves was almost completely suppressed in the *hst-21 tcu1-1* double mutant. The *imn* mutant carries a T-DNA insertion in the At5g53480 gene, which encodes a homolog of the human importin β1 [48]. The *imn* mutant exhibited generalized depigmentation, small rosettes and early flowering. The phenotype of the *tcu1-1* *imn* double mutant was synergistic, with pale, small, narrow and pointed leaves, and long petioles (Figure 6T). The IMN protein was identified as an interactor of NUP136 using mass spectrometry [20], but has not been studied further. Taken together, these observations suggest a functional relationship between TCU1 and other NPC proteins.

**Figure 7:**
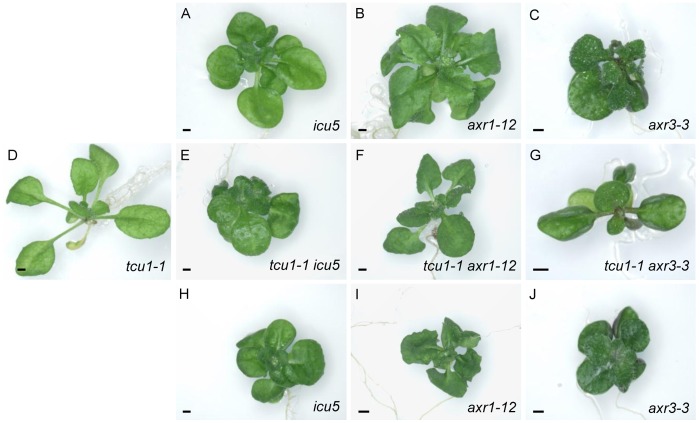
Genetic interactions between *tcu1-1* and alleles of genes encoding components of the auxin signaling pathway. The genetic backgrounds of the plants shown are (A) En-2, (B) Col-0, (C) Col-1, (D) L*er*, (E, H) L*er*/En-2, (F, I) L*er*/Col-0 and (G, J) L*er*/Col-1. All plants are homozygous for the mutations indicated. Pictures were taken at 21 das. Scale bars: 1 mm.

We also crossed *tcu1-1* to mutants carrying alleles of genes encoding components of the auxin signaling pathway. These included mutants in two Aux/IAA genes, the *axr3-3* semidominant allele of *AUXIN RESISTANT3* [49], [50], and the *icu5* dominant allele of *SHORT HYPOCOTYL2* (*SHY2*) [51]–[54]. We also crossed *tcu1-1* to the recessive *axr1-12* allele of *AXR1*, encoding a subunit of the RUB1 activating enzyme, which regulates the protein degradation activity of SCF complexes [55]. The *tcu1-1 axr3-3* and *tcu1-1 axr1-12* double homozygotes exhibited phenotypes intermediate between those of their *tcu1-1* and *axr3-3* single mutant siblings, and the *tcu1-1 icu5* and *icu5* siblings were indistinguishable (Figure 7). The observed partial mutual suppression of *tcu1-1* and *axr3-3,* as well as that of *tcu1-1* and *axr1-12* suggests that *TCU1* interacts with *AXR3* and *AXR1*. The observation that *icu5* is epistatic to *tcu1-1* also suggests an interaction between *SHY2* and *TCU1*.

### The *tcu1* Mutants are Hypersensitive to 2,4-D and Paclobutrazol and Defective in Light Perception

We further characterized the functions of TCU1 by examining the hormone responses in *tcu1-1* mutants. To examine whether TCU1 plays a role in auxin responses, we tested whether the *tcu1-1* mutant was hypersensitive to 2,4-D (2,4-dichlorophenoxyacetic acid). On medium containing 50 nM 2,4-D, root growth was reduced to 51.8% (compared to untreated roots) in the mutant and 95.05% in L*er*. In addition, the *tcu1-1* mutant was hypersensitive to the blockage of gibberellin synthesis caused by paclobutrazol: germination inhibition was stronger in *tcu1-1* (22.7% germination) than in L*er* (51.1% germination) in the presence of 1 µM paclobutrazol. We also studied the effect of different light intensities on the phenotype of *tcu1-1* and *tcu1-2* and found that it worsened under low light (40 µmol·m^−2^·s^−1^) but was almost completely suppressed by high light (130 µmol·m^−2^·s^−1^) (Figure S8).

## Discussion

Most of our current knowledge on NPC structure and function derives from studies performed in yeast and animals. The Nup54 and Nup58 components of the Nup62 subcomplex were first identified from rat liver nuclei [56]. Later, Nup54, Nup58 and Nup62 were found to coprecipitate in a complex from rat and *Xenopus* protein nuclear extracts [57]. More recently, the crystal structures of the interacting domains of *Rattus norvegicus* Nup54, Nup58 and Nup62 were determined, indicating that as many as 224 copies of these proteins form a flexible transport channel with a ring diameter that can be modulated by circumferential sliding induced by interactions with transport receptors [58], [59].

The Nup62 subcomplex has received little attention in plants. Arabidopsis NUP54, NUP58 and NUP62 coimmunoprecipitate with RAE1-GFP, a GFP-tagged nucleoporin [20]. Although insertional alleles of the corresponding genes are available, no mutational analysis has been published, the only exception being the demonstration that overexpression-based cosuppression of the gene encoding NUP62 causes stunted growth and early flowering [60].

In this work, positional cloning, complementation and comparative analysis of five *tcu1* mutant alleles confirmed that their phenotype is caused by loss of function of the At4g37130 gene, which encodes a likely ortholog of the Nup58 nucleoporin of yeast and vertebrates. All *tcu1* alleles studied here are viable and recessive and their lesions most likely cause a partial or complete loss of function of *TCU1*. *TCU1* is a single-copy gene, but does not seem to be essential in Arabidopsis, an idea supported by the viability of mutant plants carrying the *tcu1-1* allele, which encodes a truncated protein with only 61 aa. Indeed, the gene encoding NUP62 is included in the database of Arabidopsis embryonic lethal mutants, but the genes encoding TCU1/NUP58 and NUP54 are not included, indicating that they also are not essential [61]. The *TCU1_pro_:TCU1*, *2x35S_pro_:TCU1* and *2x35S_pro_:tcu1-1* transgenes did not alter the phenotype in wild type backgrounds, indicating that an excess of TCU1/NUP58 function has no significant morphological effects and suggesting that the truncated protein produced by *tcu1-1* is not antimorphic.

We have shown that *TCU1* is expressed in all the tissues and organs studied, as would be expected for a putatively domestic function that is likely to be required in every cell. The spatial expression pattern of *TCU1* is similar to those of other previously described genes encoding Arabidopsis nucleoporins, such as *NUA* [62] and *SAR1* and *SAR3* [13]. We have also shown that a functional TCU1:GFP fusion protein localizes to the nuclear envelope, in line with the results of Tamura *et al*. [20]. Perinuclear localization has also been demonstrated for other putative NPC components of Arabidopsis: SAR3/NUP96 [13], WIP1, WIP2a and WIP3 [8], NUA/TPR [7], and SEH1, SEC13, GP210, NUP54, NUP75, NUP88, NUP93a, NUP107, NUP1/NUP136, NUP160/SAR1, NUP205 and NUP214 [20].

The pleiotropic phenotype of *tcu1* mutants suggests that *TCU1* is required in several aspects of Arabidopsis development and physiology. The increased length of *tcu1-1* petioles and hypocotyls results from increased cell elongation. The size reduction of the lamina of *tcu1-1* appears to be due to a reduced number of cell divisions in both the epidermis and palisade mesophyll of vegetative leaves, whose cells show no appreciable changes in shape or size. A deficit in the number of leaf cells is particularly apparent in the spongy mesophyll, which exhibits large air spaces.

We also conducted a Y2H screen for TCU1/NUP58 interactors; this screen identified 34 genes, including 17 that encode known nuclear-localized proteins. An indicator of the quality of the screen is that one of the preys identified was NUP62. These results are consistent with our genetic interaction analyses, in which we combined alleles of *TCU1* and genes encoding members of the Nup62 and Nup107–160 nucleopore subcomplexes, an exportin and an importin. The double mutant phenotypes were synergistic in all cases, indicating a functional relationship with TCU1/NUP58 and suggesting that TCU1 acts at the nucleopore and is involved in nucleocytoplasmic trafficking.

Some hormone signals were altered in the *tcu1-1* mutant, which was hypersensitive to 2,4-D, a synthetic auxin, and paclobutrazol, an inhibitor of gibberellin synthesis. The hypersensitivity of the *tcu1-1* mutant to paclobutrazol during germination suggests insufficient translocation to the nucleus of a positive regulator of the gibberellin pathway. *AXR1* encodes an enzyme required for the activation of SCF ubiquitin ligase complexes [55], [63], [64]. Insensitivity to auxin in *axr1* mutants is due to the nuclear accumulation of Aux/IAA proteins, which regulate auxin-responsive genes and are regulated by the SCF^TIR1^ complex (reviewed in [65]. The hypersensitivity to auxin in *tcu1-1* mutants suggests that TCU1/NUP58 is required for the translocation of one or several SCF^TIR1^ complex components or Aux/IAA transcription factors to the nucleus. In line with this, Parry *et al*. [13] proposed that altered auxin homeostasis explains the phenotypes of *sar1* and *sar3* mutants. These authors hypothesized that altered NPC function impairs the nuclear import of an auxin signaling protein in *sar1* and *sar3* mutants. Although alterations in leaf vasculature are frequently found in mutants affected in auxin homeostasis, the venation pattern of the *tcu1-1* mutant was relatively normal.

The mutant phenotype of *tcu1* alleles is enhanced by reduced light intensities and suppressed by increased light. This observation suggests that TCU1/NUP58 is required for light perception. In addition, the phenotype of *tcu1* mutants includes traits that suggest an altered photomorphogenic response: early flowering, leaf lamina size reduction, increased hypocotyl and petiole length, and acute angles of the petioles with the stem. These traits define the shade avoidance syndrome shown by plants growing under a canopy of other plants, which partially deprive them of sunlight (reviewed in [66], [67]). Solar radiation is rich in the red and blue components of the visible spectrum, which are partly absorbed by the plants exposed to direct sunlight, creating an area of shadow enriched in far-red wavelengths. Light rich in the far-red component is perceived by PHYB and other photoreceptors, activation of which triggers changes in the expression levels of many genes, which in turn gives rise to the morphological changes characterizing the shade avoidance syndrome [68]–[70]. Poor perception of visible light and, in particular, of its red component might explain the constitutive shade avoidance syndrome of *tcu1* mutants, as well as its suppression by intense light.

Reduced Nup96 levels impair immune responses in mice, apparently due to reduced export of mRNAs encoding proteins regulating immunity [71]. In spite of the phylogenetic distance between mammals and plants, Nup96 and two other nucleoporins of the Nup107–160 subcomplex are also involved in immunity in Arabidopsis. A connection with defense has been demonstrated only for NUP96 (MOS3) [9], SEH1 and NUP160 [72], but not for the remaining five nucleoporins of the Nup107–160 complex, which are encoded by single-copy genes in the Arabidopsis genome and should not, in principle, be affected by redundancy. This observation supports several explanations, one of which is that some nucleoporins have specific functions, not necessarily related to their contribution to nucleocytoplasmic transport.

Comparisons among mutants affected in NPC function in Arabidopsis clearly indicate the existence of both common and specific phenotypic traits. Common traits suggest that the genes are involved in the same process. By contrast, the specific traits might be attributed to allele specificities when a single allele is available. However, two additional likely explanations are (1) that specific NPC components are particularly critical for the transport of specific molecules, and (2) that specific NPC components play roles beyond nuclear transport. The genetic and physical interactions described here are consistent with the hypothesis that TCU1/NUP58 is a member of the Nup62 subcomplex of the Arabidopsis NPC. Our findings also suggest regulatory roles for TCU1/NUP58 beyond its participation in nucleocytoplasmic trafficking. In this regard, it is worth noting experimental evidence obtained in yeast, *Drosophila melanogaster* and mammals, that indicates active role of nucleopore components in regulating gene expression. Some nucleoporins are suspected to act as insulators in human cells, separating active and inactive chromatin domains (reviewed in [73]. Studies in yeast and human cells indicate that nucleopore components are also involved in DNA repair and maintenance of genome integrity (reviewed in [74]). In addition, there are several nucleoporins of *Drosophila melanogaster* with a demonstrated function outside the nucleopore [75]: Nup88, Nup98 and Sec13 bind to chromatin and Nup98 and Sec13 act as transcription factors [76]; Nup153 is also involved in transcription [77], and Nup98, Nup62 Nup50 act together as transcription factors in embryonic cells [78].

A close relationship exists between nucleoporins and sumoylation, a process apparently not related to nucleo-cytoplasmic trafficking. Mutations in the *NUA* gene cause an increase of SUMO conjugates [7], similar to that caused by mutations in *ESD4* (*EARLY IN SHORT DAYS4*), which encodes a nuclear protease that participates in sumoylation [79], [80]. NUA and ESD4 interact in Y2H assays [7]. Mutations in the gene encoding NUP160 also increase the levels of SUMO conjugates [57].

We have not studied the relationship between TCU1/NUP58 and sumoylation. However, the most represented preys among the positive clones identified in our Y2H screen were two components of the SCF complex: SKP1/ASK1 and ASK2. Since this observation suggested a connection between the TCU1/NUP58 nucleoporin and ubiquitination, we analyzed total protein extracts and found that ubiquitin conjugate levels are not different in the *tcu1* mutants and their wild types. On the contrary, we found genetic interactions between *tcu1* alleles and alleles of genes of the ubiquitin-proteasome pathway. In conclusion, the results of our Y2H analysis suggest that TCU1/NUP58 binds ASK2 and ASK1, and the results of our genetic analyses indicate a functional relationship between TCU1/NUP58 and auxin signaling, as well as with at least one component of the ubiquitin-proteasome pathway. We did not detect, however, any difference between the *tcu1* mutants and their wild types in the levels of polyubiquitinated proteins. Further experiments will be required to ascertain the role of TCU1/NUP58 in ubiquitination.

Although we have not found any relationship between TCU1/NUP58 and proteasome components, it should be noted that two of the Arabidopsis homologs of components of the yeast TREX-2 complex, which is anchored to the NPC, interact with the Arabidopsis homolog of DSS1, which is an established proteasome component in yeast and animals. This observation suggests a link between the two complexes [19].

The *tcu1* mutants may be instrumental in revealing the relationship between an apparently domestic cellular process, nucleocytoplasmic transport, and specific aspects of plant development such as light-regulated development, leaf organogenesis and flowering. Some of the observed physical and genetic interactions will require confirmation by other experimental approaches. The identified interactors indicate a possible relationship between TCU1/NUP58 and specific biological processes, including the ACT2 and ACT7 actins and development, KNAT3, HY2 and DET3 and light perception and signaling, and GAI and the flowering-promoting pathway mediated by gibberellins. Indeed, the response to gibberellin is known to be elicited through targeted degradation of DELLA proteins by the 26S proteasome via the SCF^SLY^ complex [81].

## Supporting Information

Figure S1 **Leaf cellular phenotypes of *tcu1-1* and L*er*.** (A–D) Adaxial epidermal cells shown as (A, B) interference contrast micrographs and (C, D) diagrams. (E, F) Diagrams of abaxial epidermal cells. Stomata are only partially drawn and appear as circles in C–F. (G, H) Diagrams of cells of the palisade mesophyll subepidermal layer. (I) Boxplot distribution of cell sizes in the tissues and genotypes shown. Boxes are delimited by the first (Q1, lower hinge) and third (Q3, upper hinge) quartiles. Whiskers represent Q1–1.5·IQ (lower) and Q3+1.5·IQ (upper), where IQ = Q3– Q1. **⋄**: Mean. –: Median. ○: Extreme maximum outlier (> [Q3+3·IQ]). × : Maximum outlier. Leaves were collected at 21 das (days after stratification). Scale bars: 50 µm.
(PPTX)

Figure S2 **Venation pattern in *tcu1-1* leaves.** Diagrams were drawn from first- and third-node leaves collected 21 das. The leaf margin is shown in orange. Some excisions at the margin were required to flatten *tcu1-1* leaves before microscopy. Scale bar: 2 mm.
(PPTX)

Figure S3 **Phenotypic complementation of *tcu1-1* by the *TCU1_pro_:TCU1* transgene.** The plants shown were isolated on medium supplemented with 15 µg·ml^−1^ hygromicin among the T1 progeny of *tcu1-1* plants transformed by infection with *Agrobacterium tumefaciens* C5851 cells carrying the pGreen0179 plasmid either without any insert (left) or with the *TCU1_pro_:TCU1* insert (right). The picture was taken 26 das. Scale bar: 2 cm.
(PPTX)

Figure S4 **Comparison of deduced amino acid sequences of TCU1 and the putative Nup58s of some higher plants.** The TCU1/NUP58 protein of *Arabidopsis thaliana* (*At*; NP195430.2) is aligned with homologous gene products from *Nicotiana tabacum* (*Nt*; ACY30439.1), *Vitis vinifera* (*Vv*; XP_002282659), *Populus balsamifera* subsp. *Trichocarpa* (*Pb*; XP_002310761.1), *Oryza sativa* (*Os*; NP_001063345.1) and *Zea mays* (*Zm*; NP001132589). Amino acid residues identical or similar in all five sequences are shaded black or grey, respectively. The first of the 452 amino acids that are predicted to be missing in the *tcu1-1* mutant is shaded red. The alignment was obtained using Clustal X 2.0 (Larkin *et al*., 2007) and shaded with Boxshade 3.21 (http://www.ch.embnet.org/software/BOX_form.html).
(PPTX)

Figure S5 **Expression data output obtained from the Arabidopsis Electronic Fluorescent Pictograph (eFP) Browser for At4g37130 (*TCU1*) expression levels throughout all Arabidopsis developmental stages.**
(PPTX)

Figure S6 **Spatial expression analysis of *TCU1*.** GUS staining of *TCU1_pro_:GUS* transgenic plants in (A) roots, (B) a cotyledon, (C–I) expanding leaves and whole rosettes. Plant material was collected at the time shown in each picture (in das). Scale bars: (A–C) 0.5 mm and (D–I) 1 mm.
(PPTX)

Figure S7 **Confirmation of interactions identified in a Y2H screen with TCU1_93–513 as bait.** Yeast PBN204 cells containing three reporters (*URA3*, *lacZ*, and *ADE2*) that are under the control of different GAL promoters were used. Yeast transformants of the TCU1_93–513 bait and 2 different Arabidopsis cDNA AD libraries were spread on SD-LWU (SD without leucine, tryptophan and uracil) selection medium, which supports growth of cells with bait and prey plasmids yielding proteins interacting each other. After selecting yeast colonies on uracil-deficient media, beta-galactosidase activity was monitored. Growth of the URA+ and lacZ+ colonies on adenosine-deficient media was also tested. This three independent reporter system reduces false positives. In order to confirm the interactions found, the prey parts of the plasmids of the positive clones were amplified by PCR and reintroduced into yeast, each with either the TCU1_93–513 bait plasmid (“Bait” in the Figure) or with a negative control plasmid (“Vector” in the Figure). The 180 clones obtained in this way were tested again for *lacZ* activity (not shown) and growth on SD-LWU (central panels) and SD-LWA (right panels) media. Numbers at the left panel correspond to the clone identifiers shown in.+and −: positive and negative controls of protein-protein interaction. Image and information provided by PanBioNet.
(PPTX)

Figure S8 **Effects of light intensity on the morphological phenotype of *tcu1-1* and *tcu1-2*.** Plants were grown on plates using our standard culture conditions, under continuous light of the photon flux densities shown. Scale bars: 1 mm.
(PPTX)

Table S1 **Arabidopsis mutants used in this work.** ^a^Alternative allele names are indicated in parentheses. ^b^Sequence obtained in this work. ^c^Molecular nature of the mutation yet to be determined. ^d^Berná *et al.* (1999). ^e^SIGnAL collection (http://signal.salk.edu). ^f^SAIL collection (http://www.syngenta.com). ^g^Parry *et al*. (2006). ^h^Esteve-Bruna *et al.* (2013). ^i^Leyser *et al.* (1993). ^j^Rouse *et al.* (1998).
(DOCX)

Table S2 **Oligonucleotide sets used for the fine mapping of** ***TCU1*** **.** *Labeled with TET (4,7,2′,7′-tetrachloro-6-carboxyfluorescein).
(DOCX)

Table S3 **Other oligonucleotide sets used in this work.** ^a–e^These oligonucleotides include at their 5′ends ^a^ *EcoR*I and ^b^ *Xba*I restriction sites, ^c^the CACC sequence recognized by the vaccinia virus topoisomerase, and ^d^attB1 and ^e^attB2 sequences, which are shown in italics. ^f^One half of the primer hybridizes to the 3′ end of one exon and the other half to the 5′ end of the next exon.
(DOCX)

Table S4 **Morphometry of the venation pattern of first- and third-node** ***tcu1-1*** **leaves.** All values are means ± standard deviations from at least 10 measures.
(DOCX)

Table S5 **Results of a Y2H-based screen using TCU1_93-513 as bait.**
(DOCX)
