# Supplementary material for: Arabidopsis TRANSCURVATA1 Encodes NUP58, a Component of the Nucleopore Central Channel
Source: PLoS One. 2013 Jun 28;8(6):e67661. doi: 10.1371/journal.pone.0067661 (PMC3695937; doi:10.1371/journal.pone.0067661)
Supplement: Figure S1 — Leaf cellular phenotypes of tcu1-1 and Ler. (A–D) Adaxial epidermal cells shown as (A, B) interference contrast micrographs and (C, D) diagrams. (E, F) Diagrams of abaxial epidermal cells. Stomata are only partially drawn and appear as circles in C–F. (G, H) Diagrams of cells of the palisade mesophyll subepidermal layer. (I) Boxplot distribution of cell sizes in the tissues and genotypes shown. Boxes are delimited by the first (Q1, lower hinge) and third (Q3, upper hinge) quartiles. Whiskers represent Q1–1.5·IQ (lower) and Q3+1.5·IQ (upper), where IQ = Q3– Q1. ⋄: Mean. –: Median. ○: Extreme maximum outlier (> [Q3+3·IQ]). × : Maximum outlier. Leaves were collected at 21 das (days after stratification). Scale bars: 50 µm. (PPTX) [file pone.0067661.s001.pptx]

## Slide 1
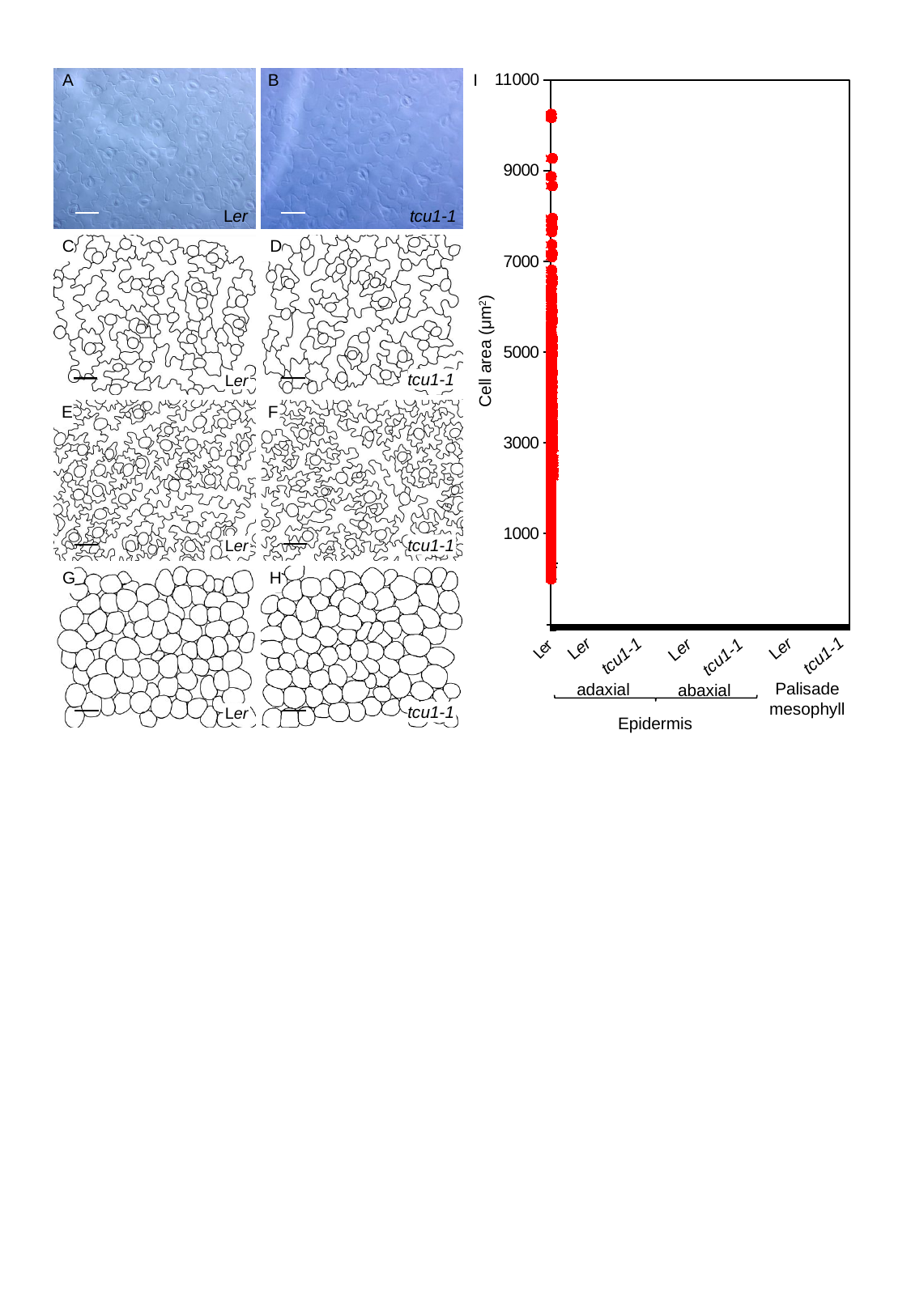

### Chart
| Category | Minimum | First Quartile | Second Quartile | Third Quartile | Maximum | Fourth Quartile - | Third Quartile - | Second Quartile - | | | | | | | | | | | | | 1 Q | 3 Q | Median | Average |
|---|---|---|---|---|---|---|---|---|---|---|---|---|---|---|---|---|---|---|---|---|---|---|---|---|
| Ler | 6.184744 | 449.24242849999996 | 462.68393850000007 | 833.0432099999999 | 1e-300 | 1e-300 | 1e-300 | 1e-300 | None | 1.148148 | 1.683998 | 1.095517 | 258.307481 | 343.525082 | None | 1.148148 | 1.683998 | 1.095517 | 258.307481 | 343.525082 | 455.4271725 | 1751.154321 | 918.111111 | 1314.32001334776 |
| tcu1-1 | 1.148148 | 430.141846 | 400.44705650000003 | 832.46357525 | 1e-300 | 1e-300 | 1e-300 | 1e-300 | 6.184744 | 7.148148 | 3.056531 | 1.938272 | 356.029429 | 348.191408 | 6.184744 | 7.148148 | 3.056531 | 1.938272 | 356.029429 | 348.191408 | 431.289994 | 1664.20062575 | 831.7370505 | 1198.1712116864157 |
| Ler | 1.683998 | 271.8795145 | 266.9875515 | 546.7091499999999 | 1e-300 | 1e-300 | 1e-300 | 1e-300 | 8.395356 | 7.689433 | 3.083203 | 5.869229 | 368.869208 | 359.42282 | 8.395356 | 7.689433 | 3.083203 | 5.869229 | 368.869208 | 359.42282 | 273.5635125 | 1087.260214 | 540.551064 | 896.4550682422861 |
| tcu1-1 | 1.095517 | 248.9901005 | 351.48401750000005 | 689.2185614999999 | 1e-300 | 1e-300 | 1e-300 | 1e-300 | 12.351129 | 10.765432 | 3.417526 | 10.234568 | 371.767384 | 376.982257 | 12.351129 | 10.765432 | 3.417526 | 10.234568 | 371.767384 | 376.982257 | 250.0856175 | 1290.7881965 | 601.5696350000001 | 1014.8063690845247 |
| Ler | 258.307481 | 750.22260325 | 284.26053224999987 | 337.2050730000001 | 1e-300 | 1e-300 | 1e-300 | 1e-300 | 13.187192 | 18.641975 | 4.148084 | 14.135802 | 377.567901 | 388.09969 | 13.187192 | 18.641975 | 4.148084 | 14.135802 | 377.567901 | 388.09969 | 1008.5300842500001 | 1629.9956895 | 1292.7906165 | 1349.9580083352434 |
| tcu1-1 | 343.525082 | 556.9388295 | 255.21761100000003 | 282.5962549999999 | 1e-300 | 1e-300 | 1e-300 | 1e-300 | 17.746987 | 24.591328 | 6.376905 | 17.209877 | 422.502839 | 400.022817 | 17.746987 | 24.591328 | 6.376905 | 17.209877 | 422.502839 | 400.022817 | 900.4639115 | 1438.2777775 | 1155.6815225 | 1201.751399497519 |A
B
I
Ler
tcu1-1
C
D
Cell area (μm2)
tcu1-1
Ler
F
E
tcu1-1
Ler
G
H
Ler
tcu1-1
Ler
tcu1-1
Ler
tcu1-1
adaxial
abaxial
Palisade mesophyll
Epidermis
tcu1-1
Ler
