# Supplementary figures and images for: Arabidopsis TRANSCURVATA1 Encodes NUP58, a Component of the Nucleopore Central Channel
Source: PLoS One. 2013 Jun 28;8(6):e67661. doi: 10.1371/journal.pone.0067661 (PMC3695937; doi:10.1371/journal.pone.0067661)

## Slide 1
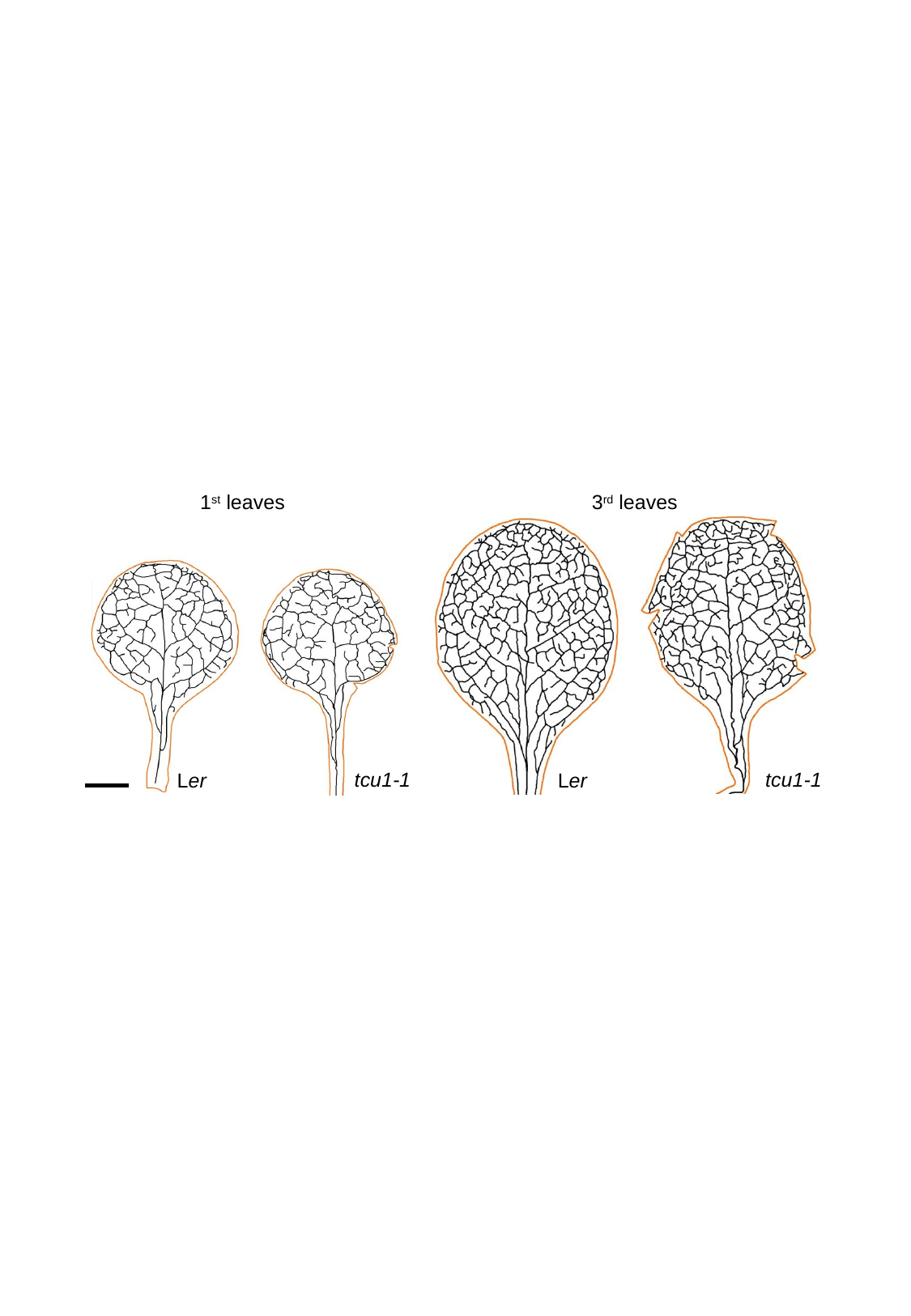

1st leaves
3rd leaves
tcu1-1
tcu1-1
Ler
Ler

Supplement: Figure S2 — Venation pattern in tcu1-1 leaves. Diagrams were drawn from first- and third-node leaves collected 21 das. The leaf margin is shown in orange. Some excisions at the margin were required to flatten tcu1-1 leaves before microscopy. Scale bar: 2 mm. (PPTX) [file pone.0067661.s002.pptx]

## Slide 1
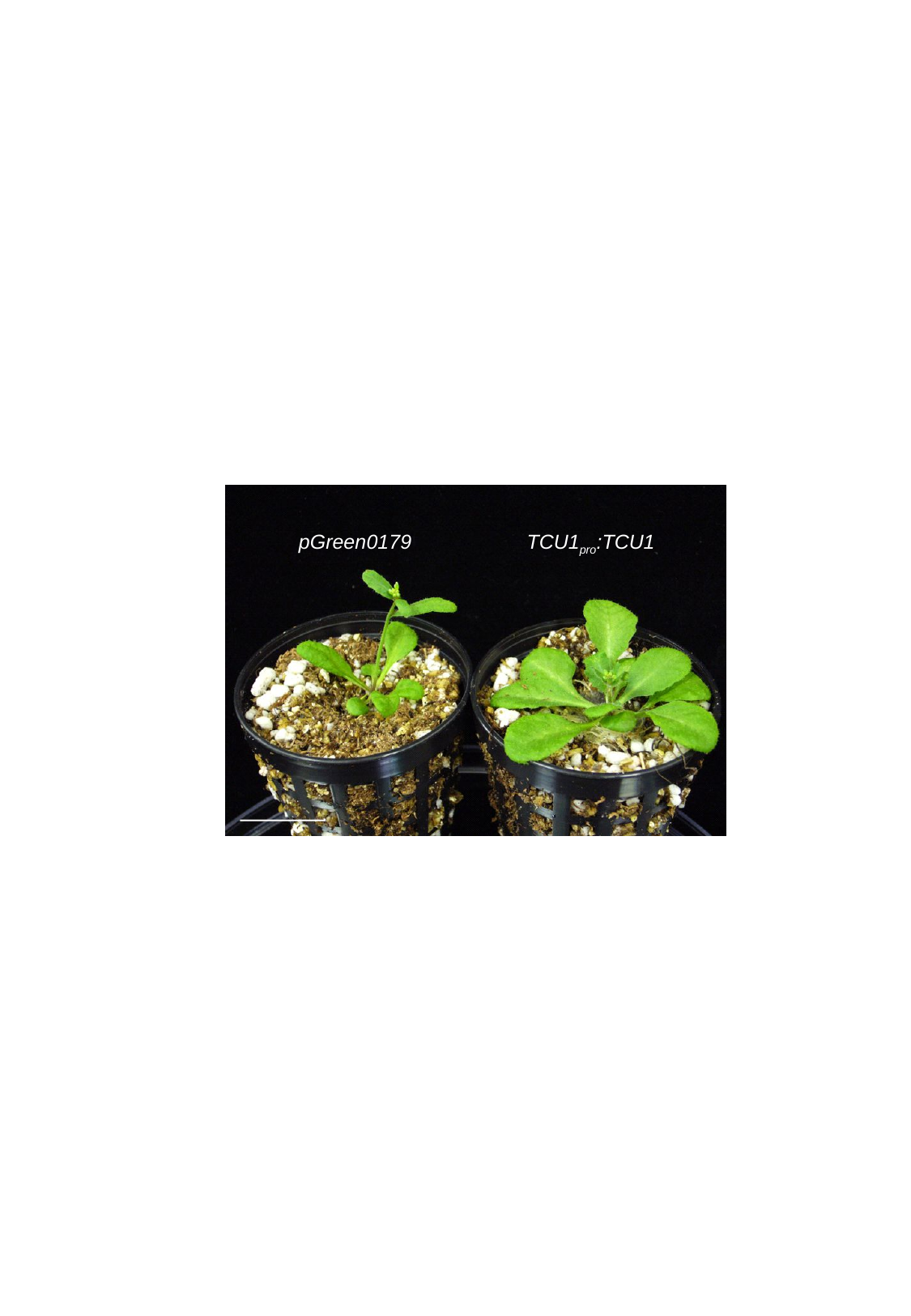

TCU1pro:TCU1
pGreen0179

Supplement: Figure S3 — Phenotypic complementation of tcu1-1 by the TCU1pro:TCU1 transgene. The plants shown were isolated on medium supplemented with 15 µg·ml−1 hygromicin among the T1 progeny of tcu1-1 plants transformed by infection with Agrobacterium tumefaciens C5851 cells carrying the pGreen0179 plasmid either without any insert (left) or with the TCU1pro:TCU1 insert (right). The picture was taken 26 das. Scale bar: 2 cm. (PPTX) [file pone.0067661.s003.pptx]

## Slide 1
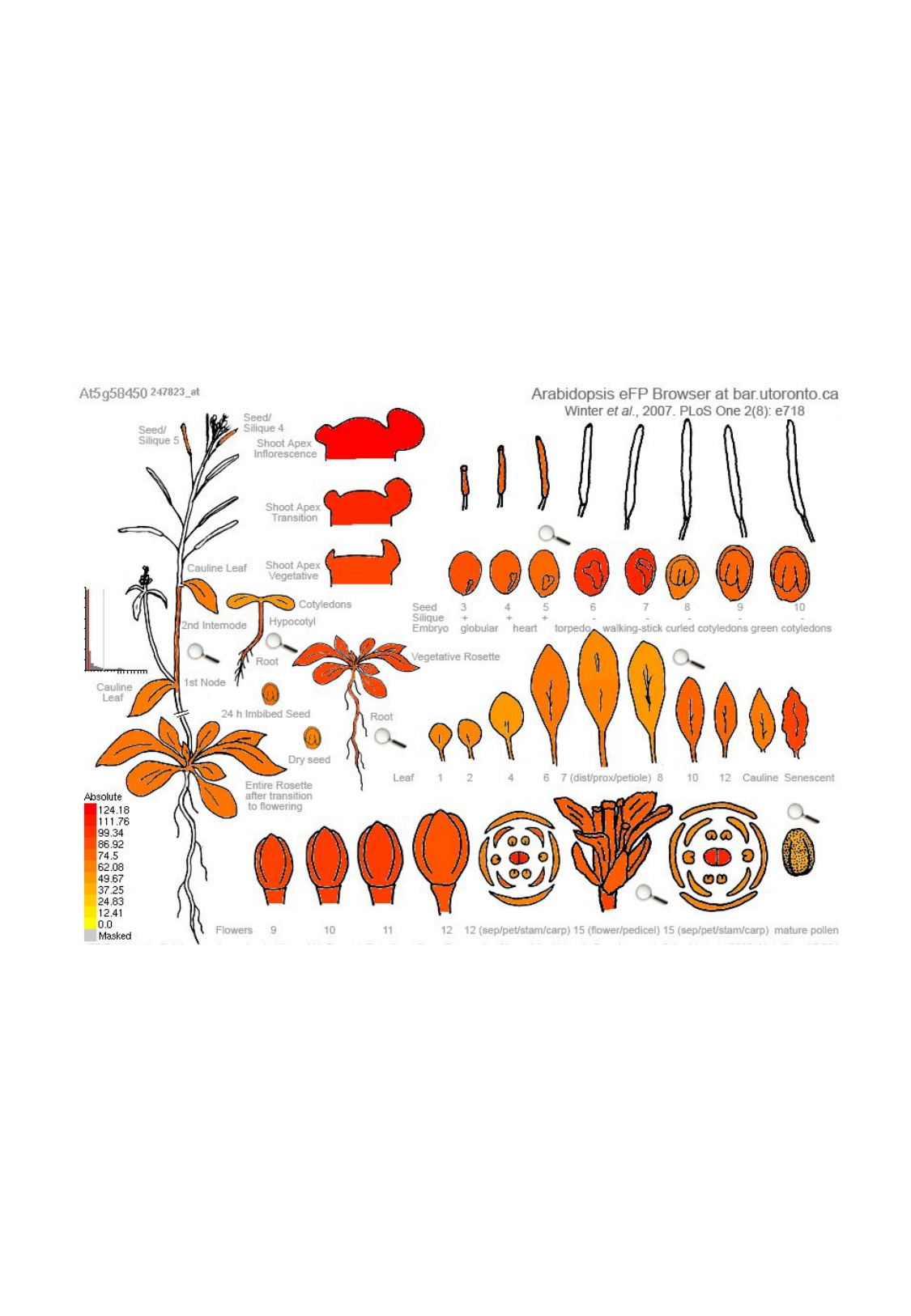

TCU1pro:TCU1
pGreen0179

Supplement: Figure S5 — Expression data output obtained from the Arabidopsis Electronic Fluorescent Pictograph (eFP) Browser for At4g37130 (TCU1) expression levels throughout all Arabidopsis developmental stages. (PPTX) [file pone.0067661.s005.pptx]

## Slide 1
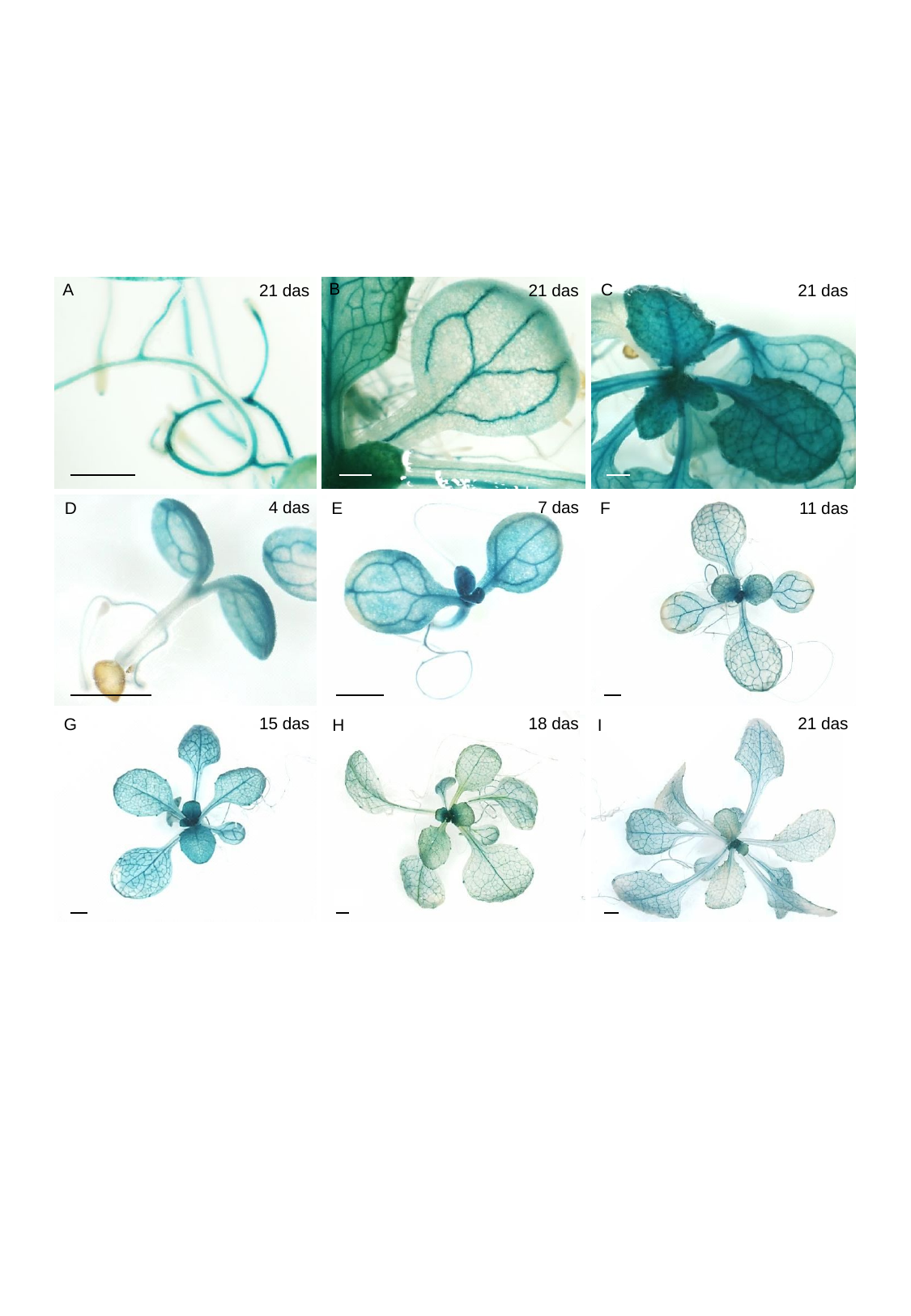

B
A
C
21 das
21 das
21 das
4 das
7 das
11 das
D
E
F
15 das
18 das
21 das
G
H
I

Supplement: Figure S6 — Spatial expression analysis of TCU1. GUS staining of TCU1pro:GUS transgenic plants in (A) roots, (B) a cotyledon, (C–I) expanding leaves and whole rosettes. Plant material was collected at the time shown in each picture (in das). Scale bars: (A–C) 0.5 mm and (D–I) 1 mm. (PPTX) [file pone.0067661.s006.pptx]
