# Supplementary material for: Arabidopsis TRANSCURVATA1 Encodes NUP58, a Component of the Nucleopore Central Channel
Source: PLoS One. 2013 Jun 28;8(6):e67661. doi: 10.1371/journal.pone.0067661 (PMC3695937; doi:10.1371/journal.pone.0067661)
Supplement: Figure S4 — Comparison of deduced amino acid sequences of TCU1 and the putative Nup58s of some higher plants. The TCU1/NUP58 protein of Arabidopsis thaliana (At; NP195430.2) is aligned with homologous gene products from Nicotiana tabacum (Nt; ACY30439.1), Vitis vinifera (Vv; XP_002282659), Populus balsamifera subsp. Trichocarpa (Pb; XP_002310761.1), Oryza sativa (Os; NP_001063345.1) and Zea mays (Zm; NP001132589). Amino acid residues identical or similar in all five sequences are shaded black or grey, respectively. The first of the 452 amino acids that are predicted to be missing in the tcu1-1 mutant is shaded red. The alignment was obtained using Clustal X 2.0 (Larkin et al., 2007) and shaded with Boxshade 3.21 (http://www.ch.embnet.org/software/BOX_form.html). (PPTX) [file pone.0067661.s004.pptx]

## Slide 1
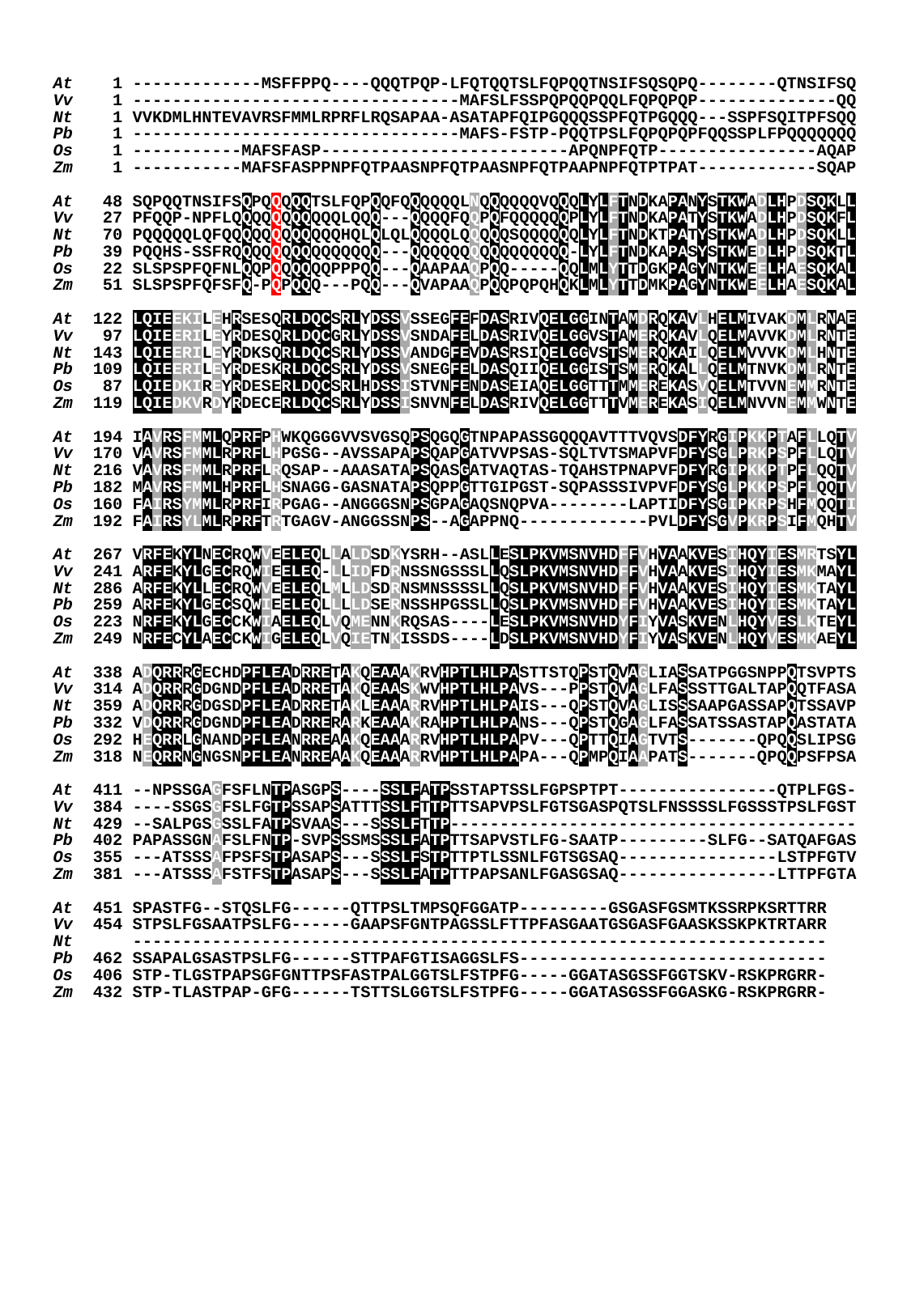

At 1 -------------MSFFPPQ----QQQTPQP-LFQTQQTSLFQPQQTNSIFSQSQPQ--------QTNSIFSQ
Vv 1 ---------------------------------MAFSLFSSPQPQQPQQLFQPQPQP--------------QQ
Nt 1 VVKDMLHNTEVAVRSFMMLRPRFLRQSAPAA-ASATAPFQIPGQQQSSPFQTPGQQQ---SSPFSQITPFSQQ
Pb 1 ---------------------------------MAFS-FSTP-PQQTPSLFQPQPQPFQQSSPLFPQQQQQQQ
Os 1 -----------MAFSFASP-------------------------APQNPFQTP----------------AQAP
Zm 1 -----------MAFSFASPPNPFQTPAASNPFQTPAASNPFQTPAAPNPFQTPTPAT------------SQAP
At 48 SQPQQTNSIFSQPQQQQQTSLFQPQQFQQQQQQLNQQQQQQVQQQLYLFTNDKAPANYSTKWADLHPDSQKLL
Vv 27 PFQQP-NPFLQQQQQQQQQQQLQQQ---QQQQFQQPQFQQQQQQPLYLFTNDKAPATYSTKWADLHPDSQKFL
Nt 70 PQQQQQLQFQQQQQQQQQQQQHQLQLQLQQQQLQQQQQSQQQQQQLYLFTNDKTPATYSTKWADLHPDSQKLL
Pb 39 PQQHS-SSFRQQQQQQQQQQQQQQQ---QQQQQQQQQQQQQQQQ-LYLFTNDKAPASYSTKWEDLHPDSQKTL
Os 22 SLSPSPFQFNLQQPQQQQQQPPPQQ---QAAPAAQPQQ-----QQLMLYTTDGKPAGYNTKWEELHAESQKAL
Zm 51 SLSPSPFQFSFQ-PQPQQQ---PQQ---QVAPAAQPQQPQPQHQKLMLYTTDMKPAGYNTKWEELHAESQKAL
At 122 LQIEEKILEHRSESQRLDQCSRLYDSSVSSEGFEFDASRIVQELGGINTAMDRQKAVLHELMIVAKDMLRNAE
Vv 97 LQIEERILEYRDESQRLDQCGRLYDSSVSNDAFELDASRIVQELGGVSTAMERQKAVLQELMAVVKDMLRNTE
Nt 143 LQIEERILEYRDKSQRLDQCSRLYDSSVANDGFEVDASRSIQELGGVSTSMERQKAILQELMVVVKDMLHNTE
Pb 109 LQIEERILEYRDESKRLDQCSRLYDSSVSNEGFELDASQIIQELGGISTSMERQKALLQELMTNVKDMLRNTE
Os 87 LQIEDKIREYRDESERLDQCSRLHDSSISTVNFENDASEIAQELGGTTTMMEREKASVQELMTVVNEMMRNTE
Zm 119 LQIEDKVRDYRDECERLDQCSRLYDSSISNVNFELDASRIVQELGGTTTVMEREKASIQELMNVVNEMMWNTE
At 194 IAVRSFMMLQPRFPHWKQGGGVVSVGSQPSQGQGTNPAPASSGQQQAVTTTVQVSDFYRGIPKKPTAFLLQTV
Vv 170 VAVRSFMMLRPRFLHPGSG--AVSSAPAPSQAPGATVVPSAS-SQLTVTSMAPVFDFYSGLPRKPSPFLLQTV
Nt 216 VAVRSFMMLRPRFLRQSAP--AAASATAPSQASGATVAQTAS-TQAHSTPNAPVFDFYRGIPKKPTPFLQQTV
Pb 182 MAVRSFMMLHPRFLHSNAGG-GASNATAPSQPPGTTGIPGST-SQPASSSIVPVFDFYSGLPKKPSPFLQQTV
Os 160 FAIRSYMMLRPRFIRPGAG--ANGGGSNPSGPAGAQSNQPVA--------LAPTIDFYSGIPKRPSHFMQQTI
Zm 192 FAIRSYLMLRPRFTRTGAGV-ANGGSSNPS--AGAPPNQ-------------PVLDFYSGVPKRPSIFMQHTV
At 267 VRFEKYLNECRQWVEELEQLLALDSDKYSRH--ASLLESLPKVMSNVHDFFVHVAAKVESIHQYIESMRTSYL
Vv 241 ARFEKYLGECRQWIEELEQ-LLIDFDRNSSNGSSSLLQSLPKVMSNVHDFFVHVAAKVESIHQYIESMKMAYL
Nt 286 ARFEKYLLECRQWVEELEQLMLLDSDRNSMNSSSSLLQSLPKVMSNVHDFFVHVAAKVESIHQYIESMKTAYL
Pb 259 ARFEKYLGECSQWIEELEQLLLLDSERNSSHPGSSLLQSLPKVMSNVHDFFVHVAAKVESIHQYIESMKTAYL
Os 223 NRFEKYLGECCKWIAELEQLVQMENNKRQSAS----LESLPKVMSNVHDYFIYVASKVENLHQYVESLKTEYL
Zm 249 NRFECYLAECCKWIGELEQLVQIETNKISSDS----LDSLPKVMSNVHDYFIYVASKVENLHQYVESMKAEYL
At 338 ADQRRRGECHDPFLEADRRETAKQEAAAKRVHPTLHLPASTTSTQPSTQVAGLIASSATPGGSNPPQTSVPTS
Vv 314 ADQRRRGDGNDPFLEADRRETAKQEAASKWVHPTLHLPAVS---PPSTQVAGLFASSSTTGALTAPQQTFASA
Nt 359 ADQRRRGDGSDPFLEADRRETAKLEAAARRVHPTLHLPAIS---QPSTQVAGLISSSAAPGASSAPQTSSAVP
Pb 332 VDQRRRGDGNDPFLEADRRERARKEAAAKRAHPTLHLPANS---QPSTQGAGLFASSATSSASTAPQASTATA
Os 292 HEQRRLGNANDPFLEANRREAAKQEAAARRVHPTLHLPAPV---QPTTQIAGTVTS-------QPQQSLIPSG
Zm 318 NEQRRNGNGSNPFLEANRREAAKQEAAARRVHPTLHLPAPA---QPMPQIAAPATS-------QPQQPSFPSA
At 411 --NPSSGAGFSFLNTPASGPS----SSLFATPSSTAPTSSLFGPSPTPT----------------QTPLFGS-
Vv 384 ----SSGSGFSLFGTPSSAPSATTTSSLFTTPTTSAPVPSLFGTSGASPQTSLFNSSSSLFGSSSTPSLFGST
Nt 429 --SALPGSGSSLFATPSVAAS---SSSLFTTP-----------------------------------------
Pb 402 PAPASSGNAFSLFNTP-SVPSSSMSSSLFATPTTSAPVSTLFG-SAATP---------SLFG--SATQAFGAS
Os 355 ---ATSSSAFPSFSTPASAPS---SSSLFSTPTTPTLSSNLFGTSGSAQ----------------LSTPFGTV
Zm 381 ---ATSSSAFSTFSTPASAPS---SSSLFATPTTPAPSANLFGASGSAQ----------------LTTPFGTA
At 451 SPASTFG--STQSLFG------QTTPSLTMPSQFGGATP---------GSGASFGSMTKSSRPKSRTTRR
Vv 454 STPSLFGSAATPSLFG------GAAPSFGNTPAGSSLFTTPFASGAATGSGASFGAASKSSKPKTRTARR
Nt ----------------------------------------------------------------------
Pb 462 SSAPALGSASTPSLFG------STTPAFGTISAGGSLFS-------------------------------
Os 406 STP-TLGSTPAPSGFGNTTPSFASTPALGGTSLFSTPFG-----GGATASGSSFGGTSKV-RSKPRGRR-
Zm 432 STP-TLASTPAP-GFG------TSTTSLGGTSLFSTPFG-----GGATASGSSFGGASKG-RSKPRGRR-
