# Supplementary material for: Arabidopsis TRANSCURVATA1 Encodes NUP58, a Component of the Nucleopore Central Channel
Source: PLoS One. 2013 Jun 28;8(6):e67661. doi: 10.1371/journal.pone.0067661 (PMC3695937; doi:10.1371/journal.pone.0067661)
Supplement: Figure S7 — Confirmation of interactions identified in a Y2H screen with TCU1_93–513 as bait. Yeast PBN204 cells containing three reporters (URA3, lacZ, and ADE2) that are under the control of different GAL promoters were used. Yeast transformants of the TCU1_93–513 bait and 2 different Arabidopsis cDNA AD libraries were spread on SD-LWU (SD without leucine, tryptophan and uracil) selection medium, which supports growth of cells with bait and prey plasmids yielding proteins interacting each other. After selecting yeast colonies on uracil-deficient media, beta-galactosidase activity was monitored. Growth of the URA+ and lacZ+ colonies on adenosine-deficient media was also tested. This three independent reporter system reduces false positives. In order to confirm the interactions found, the prey parts of the plasmids of the positive clones were amplified by PCR and reintroduced into yeast, each with either the TCU1_93–513 bait plasmid (“Bait” in the Figure) or with a negative control plasmid (“Vector” in the Figure). The 180 clones obtained in this way were tested again for lacZ activity (not shown) and growth on SD-LWU (central panels) and SD-LWA (right panels) media. Numbers at the left panel correspond to the clone identifiers shown in.+and −: positive and negative controls of protein-protein interaction. Image and information provided by PanBioNet. (PPTX) [file pone.0067661.s007.pptx]

## Slide 1
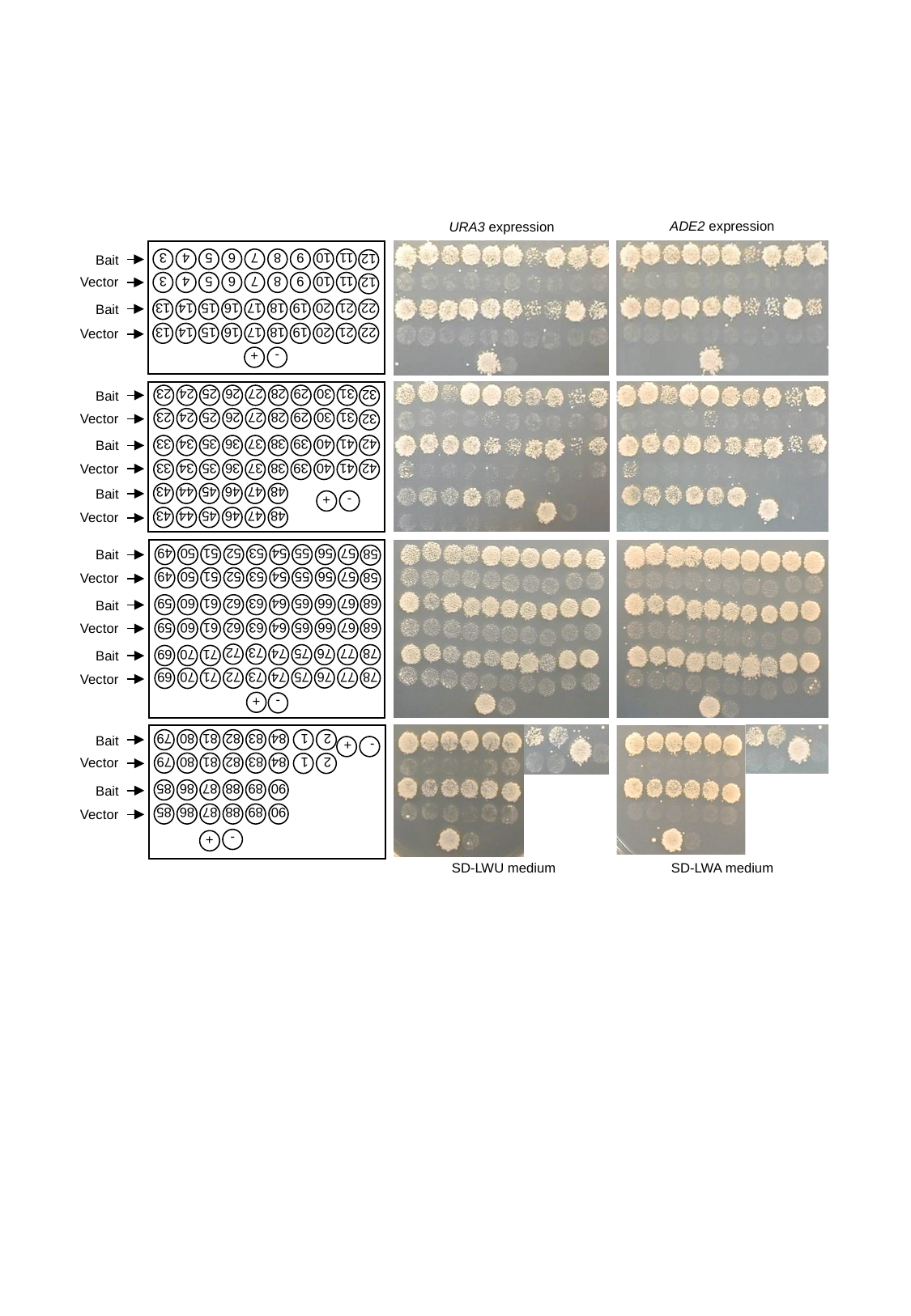

ADE2 expression
URA3 expression
Bait
4
9
10
3
5
6
7
11
8
12
Vector
4
9
10
3
5
6
7
11
8
12
Bait
22
14
19
20
13
15
16
17
21
18
Vector
22
14
19
20
13
15
16
17
21
18
+
-
Bait
24
29
30
23
25
26
27
31
28
32
Vector
24
29
30
23
25
26
27
31
28
32
Bait
42
34
39
40
33
35
36
37
41
38
Vector
42
34
39
40
33
35
36
37
41
38
Bait
44
43
45
46
47
48
+
-
Vector
44
43
45
46
47
48
Bait
50
55
56
49
51
52
53
57
54
58
Vector
50
55
56
49
51
52
53
57
54
58
Bait
68
60
65
66
59
61
62
63
67
64
Vector
68
60
65
66
59
61
62
63
67
64
Bait
72
73
75
74
76
77
78
71
69
70
Vector
75
74
76
77
78
72
73
71
69
70
+
-
Bait
2
80
1
79
81
82
83
84
-
+
tcu1-1
Vector
80
79
81
82
83
84
2
1
Bait
86
85
87
88
89
90
Vector
86
85
87
88
89
90
-
+
SD-LWU medium
SD-LWA medium
