# Supplementary material for: Arabidopsis TRANSCURVATA1 Encodes NUP58, a Component of the Nucleopore Central Channel
Source: PLoS One. 2013 Jun 28;8(6):e67661. doi: 10.1371/journal.pone.0067661 (PMC3695937; doi:10.1371/journal.pone.0067661)
Supplement: Table S1 — Arabidopsis mutants used in this work. aAlternative allele names are indicated in parentheses. bSequence obtained in this work. cMolecular nature of the mutation yet to be determined. dBerná et al. (1999). eSIGnAL collection (http://signal.salk.edu). fSAIL collection (http://www.syngenta.com). gParry et al. (2006). hEsteve-Bruna et al. (2013). iLeyser et al. (1993). jRouse et al. (1998). (DOCX) [file pone.0067661.s009.docx]

| \| **Table S1.** Arabidopsis mutants used in this work \| \| \| \| \| \| \| \| --- \| --- \| --- \| --- \| --- \| --- \| --- \| \| Alelle^a^ \| Line (NASC stock code) \| Background \| Mutagen, mutation and its effects \| Mutated gene \| Protein/alternative names \| Origin \| \| *tcu1-1* \|  \| L*er* \| EMS (C→T; Gln→Stop) \| At4g37130 \| TCU1/NUP58 \| d \| \| *tcu1-2* \| SALK_099638 (N599638) \| Col-0 \| T-DNA in the 1^st^ of 5 introns \| At4g37130 \| TCU1/NUP58 \| e \| \| *tcu1-3* \| SALK_023793 (N523793) \| Col-0 \| T-DNA in the 1^st^ of 6 exons \| At4g37130 \| TCU1/NUP58 \| e \| \| *tcu1-4* \| SAIL_655_C09 (N828449) \| Col-0 \| T-DNA in the 1^st^ of 6 exons \| At4g37130 \| TCU1/NUP58 \| f \| \| *tcu1-5* \| SAIL_349_B01 (N816242) \| Col-0 \| T-DNA in the 2^nd^ of 6 exons \| At4g37130 \| TCU1/NUP58 \| f \| \| *nup54-1* \| SALK_106346 (N606346) \| Col-0 \| T-DNA in the 1^st^ of 9 exons \| At1g24310 \| NUP54 \| e \| \| *nup54-2* \| SALK_015252 (N658102) \| Col-0 \| T-DNA in the 7^th^ of 8 introns \| At1g24310 \| NUP54 \| e \| \| *nup62-1* \| SALK_037337 (N537337) \| Col-0 \| T-DNA in the 5^th^ of 8 introns \| At2g45000 \| NUP62 \| e \| \| *nup62-2* \| SAIL_127_F01 (N806197) \| Col-0 \| T-DNA in the 8^th^ of 9 exons \| At2g45000 \| NUP62 \| f \| \| *sar1-4* \| SALK_126801 (N626801) \| Col-0 \| T-DNA in the 17^th^ of 26 exons \| At1g33410 \| NUP160/SAR1 \| g \| \| *sar1-5* \| SALK_133728 (N660612) \| Col-0 \| T-DNA in the 7^th^ of 26 exons \| At1g33410 \| NUP160/SAR1 \| e \| \| *sar3-1* \|  \| Col-0 \| γ-rays (point deletion) \| At1g80680 \| NUP96/SAR3/MOS3 \| g \| \| *sar3-3* \| SALK_109959 (N609959) \| Col-0 \| T-DNA in the 4^th^ of 5 introns \| At1g80680 \| NUP96/SAR3/MOS3 \| g \| \| *imn* \| SAIL_378_F08 (N817444) \| Col-0 \| T-DNA in the 3^rd^ of 3 exons \| At5g53480 \| IMN \| f \| \| *hst-21* (*icu3*) \|  \| L*er* \| EMS (G→A; Trp→Stop)^b^ \| At3g05040 \| HST \| d \| \| *as1-13* \|  \| L*er* \| EMS^c^ \| At2g37630 \| AS1 \| d \| \| *as2-11* \|  \| L*er* \| EMS^c^ \| At1g65620 \| AS2 \| d \| \| *icu5* (*shy2-10*) \| N379 \| En-2 \| Unknown (G→A; Gly→Gln) \| At1g04240 \| SHY2/IAA3/ICU5 \| h \| \| *axr1-12* \| N3076 \| Col-0 \| EMS (single nucleotide substitution at position 1426 in exon 11) \| At1g05180 \| AXR1 \| i \| \| *axr3-3* \| N57505 \| Col-1 \| EMS (T→G; Val→Gly) \| At1g04250 \| AXR3/IAA17 \| j \| |
| --- | --- | --- | --- | --- | --- | --- | --- | --- | --- | --- | --- | --- | --- | --- | --- | --- | --- | --- | --- | --- | --- | --- | --- | --- | --- | --- | --- | --- | --- | --- | --- | --- | --- | --- | --- | --- | --- | --- | --- | --- | --- | --- | --- | --- | --- | --- | --- | --- | --- | --- | --- | --- | --- | --- | --- | --- | --- | --- | --- | --- | --- | --- | --- | --- | --- | --- | --- | --- | --- | --- | --- | --- | --- | --- | --- | --- | --- | --- | --- | --- | --- | --- | --- | --- | --- | --- | --- | --- | --- | --- | --- | --- | --- | --- | --- | --- | --- | --- | --- | --- | --- | --- | --- | --- | --- | --- | --- | --- | --- | --- | --- | --- | --- | --- | --- | --- | --- | --- | --- | --- | --- | --- | --- | --- | --- | --- | --- | --- | --- | --- | --- | --- | --- | --- | --- | --- | --- | --- | --- | --- | --- | --- | --- | --- | --- | --- | --- | --- | --- | --- | --- | --- | --- | --- |
