# Supplementary material for: Arabidopsis TRANSCURVATA1 Encodes NUP58, a Component of the Nucleopore Central Channel
Source: PLoS One. 2013 Jun 28;8(6):e67661. doi: 10.1371/journal.pone.0067661 (PMC3695937; doi:10.1371/journal.pone.0067661)
Supplement: Table S2 — Oligonucleotide sets used for the fine mapping of TCU1 . *Labeled with TET (4,7,2′,7′-tetrachloro-6-carboxyfluorescein). (DOCX) [file pone.0067661.s010.docx]

**Table S2.** Oligonucleotide sets used for the fine mapping of *TCU1*

| Marker | | BAC clone | Oligonucleotide sequences (5´→3´) | |  | PCR product size (pb) | |
| --- | --- | --- | --- | --- | --- | --- | --- |
| Name | Type |  | Forward primer | Reverse primer |  | L*er* | Col-0 |
| F8D20 | In/Del | F8D20 | ATGGTGAAGGGCTTGTTTCAAG | CTTTTCACATTTTCCCACTCAAG |  | 319 | 403 |
| AP22 | In/Del | AP22 | GCTAGCTTCCCTAATTATGTGG | GTCCACTGTTCAATGAATTCGG |  | 394 | 443 |
| AP21 | In/Del | AP22 | TCTAGGTCCCCATCTAGGTC | TCTCTTCAGTGCCTTGACCTG |  | 213 | 194 |
| CER448306 | In/Del | AP22 | CATGATTAGCACGAAATATGTATG | CGTGATTAAGTTTCATCAGCG |  | 268 | 271 |
| CER448305 | In/Del | AP22 | TTACCTGATTCAGGAGAAATTGG | CCCAAAATCGAGCCTTGTCG |  | 208 | 211 |
| AP2 | SNP | AP21 | TCACTTTGCATAAGTGTGGTCG | GCCGGAAACAGTGAGAATCC |  | 916 | 916 |
| F6G17 | In/Del | F6G17 | CATTAACTAGTGACATTCTGTG | GGAGGTGGAGGGAGATTGAG |  | 242 | 225 |
| T28I19 | In/Del | T28I19 | CTAAACCGCTCAAGACTTCGG | CATTGACGGCTCATCTTGATTAT |  | 498 | 530 |
| nga1107 | SSLP | T9A14 | GCGAAAAAACAAAAAAATCCA | CGACGAATCGACAGAATTAGG* |  | 132 | 154 |
