# Supplementary material for: Arabidopsis TRANSCURVATA1 Encodes NUP58, a Component of the Nucleopore Central Channel
Source: PLoS One. 2013 Jun 28;8(6):e67661. doi: 10.1371/journal.pone.0067661 (PMC3695937; doi:10.1371/journal.pone.0067661)
Supplement: Table S3 — Other oligonucleotide sets used in this work. a–eThese oligonucleotides include at their 5′ends a EcoRI and b XbaI restriction sites, cthe CACC sequence recognized by the vaccinia virus topoisomerase, and dattB1 and eattB2 sequences, which are shown in italics. fOne half of the primer hybridizes to the 3′ end of one exon and the other half to the 5′ end of the next exon. (DOCX) [file pone.0067661.s011.docx]

| **Table S3.** Other oligonucleotide sets used in this work | | | |
| --- | --- | --- | --- |
| Purpose | Template | Oligonucleotide sequences (5’→ 3’) | |
| Candidate gene sequencing | At4g37130 | CCAATGAGAAATTTCCCCGGC | GAAAAAGGAAACGAATTTAGGCG |
|  | At4g37130 | atgtattgtttgcaaaagagccc | AAGCATGTCGACTATACTTGTC |
|  | At4g37130 | CCATCTCAGGGGCAAGGAAC | TCAAGCCATGTACACGATGCG |
|  | At4g37130 | TCAGAAACTCTTGCTTCAGATTG | AAACAAAGACGAGGTAGGAGC |
|  | At4g37170 | tgtggaaccttcgaagtcagg | GTGTACATATCTACAAGAGAGC |
|  | At4g37170 | GTCTAGTAGATGGCGGGAAG | gtagtagaaataaatcatagtggc |
|  | At4g37180 | GTGAGTGTAACGATTCGCCG | GGCCCTCCAAGCCTATGAAG |
|  | At4g37180 | CAATAAACCTTCTTCACAGAGTC | TCTCAGATTACGTAGATTTGTAG |
|  | At4g37190 | AAATCTAGGCTCGACGAGGG | TCTTCCACGAAAAAACGGAGTC |
|  | At4g37190 | GAATTGAACGGATTATGGATGG | CCAAAAATGGCAAAATTGCGC |
|  | At4g37190 | GAATTGAACGGATTATGGATGG | AATATCAACTCTCTCAAGCGAC |
|  | At4g37200 | CGTGTGGCTCTAATAGCTTGG | TTAGAAACAACTTCTATCATCTGC |
|  | At4g37260 | TACTCTCTCTTCTTGCTCTTATC | TAAACCCAACACGACGGCTG |
|  | At4g37260 | TACTCTCTCTTCTTGCTCTTATC | TTTCCTCGTAACAAACCCATAG |
|  | At4g37540 | TTTGTGTCAATATGACCCATTC | TCATCCCATTTTCCCAACACG |
|  | At4g37540 | GGTCGTGCTGGTCTCATGTC | TGCATTAGGATGGTGGTAGTAG |
| Double mutant genotyping | *NUP62* | GCTTCATCTACCACTTCTCCG | AAAGATGGATGATGAGGAGCC |
|  | *NUP62* | ATAGCAGAGTGGGATAAGCGG | TCCAGAAGTGATTTCCGCTCG |
|  | *NUP62* | ATAGCAGAGTGGGATAAGCGG | ATTCTTATCAAGACATCCAGTGC |
|  | *SAR3* | GTATAATCGAGAAACAGCTGG | TGAAGAACAACGTAGATAGCCC |
|  | *SAR3* | GCCAGGTTTGGGAGATCCCG | TCAGCCATCTTTGAGTATGCGAC |
|  | *SAR1* | ACTTTAGTTCTGCGGAGGGG | AAATTCAAAGCTACAGCGGACC |
|  | *SAR1* | TAGTTAACATCCTTGAGTCGGG | TTTTACTATACCGAATCCAGCTG |
|  | *NUP54* | ATACATGGACTAGGGGACATAG | TACTGTGGTTCAGTCACACTG |
|  | *NUP54* | GAAAGCTCTGCTCCAGGACC | GATCTCTCTCAGCGCCTTAAG |
| Double mutant genotyping | *IMN* | tcgcaccccatatcctccag | AAGAGAAGATAGACAGACATGTG |
| *hst-21* sequencing and genotyping | *HST* | GATTCTGCAAGACGAGTTGCA | GAGTCATCAATACCAATTCAGC |
|  | *HST* | CTTCATTGTCTGCTCAGGGCC | ACCTTCTTCACTGAAAACATCC |
|  | *HST* | ATGTGTGAGAGTATGGCCTCT | CACTGCTACATGCTGAGAATG |
|  | *HST* | tcgagcacttgttttcctctg | AACATGACAAATCTGAAGCAGG |
|  | *HST* | GCATAACATTCTGGGTGAAGC | TGATCCACTTTGTATGCCAAAC |
|  | *HST* | GTCATGGCTGGGAGTGCTTC | CTGCTTCTCTACTATTACATGG |
| Phenotypic rescue | *TCU1* | *GAATTC*CAAAGATGTTTCATCGGGGTG^a^ | *TCTAGA*CTTGTTTCTCTGCCCTGCAAG^b^ |
|  | pGreen0179 | TCTTCGCTATTACGCCAGCTG | GCGGATAACAATTTCACACAGG |
| GUS assays | *TCU1* | *CACC*TAAATGTCTTTTCCCTTTCACCA^c^ | TGCTGGATATAACCGACGGAG |
|  | pMDC164 | AAGACTGTAACCACGCGTCTG | TTGACTGCCTCTTCGCTGTAC |
| Subcellular localization | *TCU1* | *GGGGACAAGTTTGTACAAAAAAGCAGGCT*TAAATGTCTTTTCCCTTTCACCA^d^ | *GGGGACCACTTTGTACAAGAAAGCTGGGT*ACGGCGTGTAGTTCGAGATTTT^e^ |
|  | pMDC111 |  | TATGTTGCATCACCTTCACCCT |
| Constitutive expression | *TCU1* | *GGGGACAAGTTTGTACAAAAAAGCAGGCT*ATGTCGTTTTTTCCCCCACAG^d^ | *GGGGACCACTTTGTACAAGAAAGCTGGGT*CTAACGGCGTGTAGTTCGAGA^e^ |
|  | pMDC32 | TTCATTTGGAGAGGACCTCG | GAAATTCGAGCTCCACCGCG |
| qRT-PCR | *TCU1* | *CACC*ATGTCGTTTTTTCCCCCACAG^c^ | ATGCTCCAATATCTTCTCTTCAAT^f^ |
|  | *OTC* | TGAAGGGACAAAGGTTGTGTATGTT | CGCAGACAAAGTGGAATGGA |
