# Supplementary material for: Arabidopsis TRANSCURVATA1 Encodes NUP58, a Component of the Nucleopore Central Channel
Source: PLoS One. 2013 Jun 28;8(6):e67661. doi: 10.1371/journal.pone.0067661 (PMC3695937; doi:10.1371/journal.pone.0067661)
Supplement: Table S4 — Morphometry of the venation pattern of first- and third-node tcu1-1 leaves. All values are means ± standard deviations from at least 10 measures. (DOCX) [file pone.0067661.s012.docx]

**Table S4.** Morphometry of the venation pattern of first- and third-node *tcu1-1* leaves

|  |  | L*er* | *tcu1-1* | p value |
| --- | --- | --- | --- | --- |
| Leaf 1 | Area (mm^2^) | 38.19 ± 6.09 | 32.73 ± 10.07 | 1.6·10^-1^ |
|  | Venation length (mm) | 113.70 ± 15.41 | 93.76 ± 26.99 | 5.75·10^-2^ |
|  | Vascular density (mm/mm^2^) | 2.99 ± 0.16 | 2.93 ± 0.43 | 6.54·10^-1^ |
|  | Isolated veins | 0.20 ± 0.42 | 0.30 ± 0.48 | 4.71·10^-1^ |
|  | Free-ending veins | 51.40 ± 7.95 | 47.20 ± 16.19 | 6.28·10^-1^ |
|  | Branching points per mm^2^ | 137.80 ± 15.62 | 103.70 ± 30.93 | 6.02·10^-3^ |
| Leaf 3 | Area (mm^2^) | 69.01 ± 13.59 | 56.30 ± 9.21 | 2.48·10^-2^ |
|  | Venation length (mm) | 230.02 ± 26.92 | 177.91 ± 29.06 | 5.88·10^-4^ |
|  | Vascular density (mm/mm^2^) | 3.39 ± 0.33 | 3.18 ± 0.33 | 1.68·10^-1^ |
|  | Isolated veins | 0.30 ± 0.67 | 3.00 ± 2.37 | 5.25·10^-1^ |
|  | Free-ending veins | 88.10 ± 12.49 | 94.00 ± 25.89 | 8.46·10^-3^ |
|  | Branching points per mm^2^ | 283.00 ± 22.71 | 208.40 ± 37.82 | 4.4·10^-5^ |
