# Supplementary material for: Arabidopsis TRANSCURVATA1 Encodes NUP58, a Component of the Nucleopore Central Channel
Source: PLoS One. 2013 Jun 28;8(6):e67661. doi: 10.1371/journal.pone.0067661 (PMC3695937; doi:10.1371/journal.pone.0067661)
Supplement: Table S5 — Results of a Y2H-based screen using TCU1_93-513 as bait. (DOCX) [file pone.0067661.s013.docx]

| **Table S5.** Results of a Y2H-based screen using TCU1_93-513 as bait | |
| --- | --- |
| Prey identifier | Description: The activation domain (AD) is fused |
| 1,2 | in frame to the 25^th^ aa of VPS28-1 (VACUOLAR PROTEIN SORTING-ASSOCIATED PROTEIN 28 HOMOLOG 1); transporter (VPS28-1) (NM_202856) |
| 3-16 | in frame to the 58^th^ aa of ASK2 (ARABIDOPSIS SKP1-LIKE 2); protein binding/ubiquitin-protein ligase (ASK2) (NM_123584) |
| 17 | in frame to the 7^th^ aa of SKP1 (S PHASE KINASE-ASSOCIATED PROTEIN 1); protein binding/ubiquitin-protein ligase (SKP1) (NM_106245) |
| 18 | in frame to the 46^th^ aa of SKP1 |
| 19 | in frame to the 72^nd^ aa of SKP1 |
| 20, 21 | in frame to the 89^th^ aa of SKP1 |
| 22 | in frame to the 89^th^ aa of DET3 (DE-ETIOLATED 3); proton-transporting ATPase, rotational mechanism (DET3) (NM_101154) |
| 23 | in frame to the 130^th^ aa of DET3 |
| 24, 25 | in frame to the 202^nd^ aa of ACT7 (ACTIN 7); structural constituent of cytoskeleton (ACT7) (NM_121018) |
| 26, 27 | to the 5’ UTR of neighbor of GAI (GIBBERELLIC ACID INSENSITIVE); transcription factor (GAI), mRNA at -97 nt (NM_101361) |
| 28-30 | in frame to the 557^th^ aa of EMB2766 (EMBRYO DEFECTIVE 2766); structural constituent of nuclear pore (EMB2766) (NM_180098) |
| 31 | to the 5’ UTR of neighbor of PAP1 (PRODUCTION OF ANTHOCYANIN PIGMENT 1); DNA binding / transcription factor (PAP1), mRNA at -9 nt (NM_104541) |
| 32 | in frame to the 24^th^ aa of NADH-ubiquinone oxidoreductase 23 kDa subunit, mitochondrial (TYKY) (AT1G79010) (NM_106551) |
| 33-36 | in frame to the 891^st^ aa of unknown protein (AT3G20720) (NM_112963) |
| 37, 38 | to the 5’ UTR of neighbor of unknown protein (AT1G79160) mRNA, complete cds, mRNA at -12 nt (NM_106567) |
| 39, 40 | in frame to the 151^st^ aa of mutase family protein (AT1G21440) (NM_101995) |
| 41 | to the 5’ UTR of transcriptional coactivator (AT5G03500), mRNA at -35 nt (NM_120430) |
| 42 | in frame to the 3^rd^ aa of unknown protein (AT4G26410) (NM_118774) |
| 43 | in frame to the 603^rd^ aa of unknown protein (AT5G58510) (NM_125237) |
| 44, 45 | in frame to the 640^th^ aa of FTSH1 (FtsH protease 1); ATP-dependent peptidase/ ATPase/ metallopeptidase (FTSH1) (NM_103909) |
| 46 | in frame to the 416^th^ aa of RHM1 (RHAMNOSE BIOSYNTHESIS 1); UDP-L-rhamnose synthase/ UDP-glucose 4,6-dehydratase/ catalytic (RHM1) (NM_106504) |
| 47 | in frame to the 202^nd^ aa of ACT2 (ACTIN 2); structural constituent of cytoskeleton (ACT2) (NM_112764) |
| 48 | in frame to the 496^th^ aa of transporter (AT5G56160) (NM_124998) |
| 49-51 | in frame to the 94^th^ aa of PRXCB (PEROXIDASE CB); peroxidase (PRXCB) (NM_114771) |
| 52 | in frame to the 130^th^ aa of DET3 |
| 53 | to the 5’ UTR of neighbor of GAI |
| 54-57 | in frame to the 140^th^ aa of GAI |
| 58 | in frame to the 208^th^ aa of GAI |
| 59 | in frame to the 276^th^ aa of unknown protein (AT3G54630) (NM_115320) |
| 60 | in frame to the 557^th^ aa of EMB2766 |
| 61 | in frame to the 58^th^ aa of ASK2 |
| 62 | in frame to the 72^nd^ aa of SKP1 |
| 63, 64 | in frame to the 89^th^ aa of SKP1 |
| 65 | in frame to the 146^th^ aa of unknown protein (AT3G05270) (NM_001125107) |
| 66-69 | in frame to the 891^st^ aa of unknown protein (AT3G20720) |
| 70 | in frame to the 27^th^ aa of KNAT3 (KNOTTED1-LIKE HOMEOBOX GENE 3); transcription activator/ transcription factor (KNAT3) (NM_001036861) |
| 71 | in frame to the 197^th^ aa of ACT7 |
| 72 | in frame to the 202^nd^ aa of ACT7 |
| 73 | in frame to the 361^st^ aa of SHM4 (serine hydroxymethyltransferase 4); catalytic/ glycine hydroxymethyltransferase/ pyridoxal phosphate binding (SHM4) (NM_117467) |
| 74, 75 | in frame to the 204^th^ aa of HY2 (ELONGATED HYPOCOTYL 2); phytochromobilin:ferredoxin oxidoreductase (HY2) (NM_180665) |
| 76 | in frame to the 3^rd^ aa of unknown protein (AT4G26410) |
| 77, 78 | in frame to the 151^st^ aa of mutase family protein (AT1G21440) (NM_101995) |
| 79 | to the 5’ UTR of neighbor of mRNA for hypothetical protein, mRNA at -6 nt (AK230337) |
| 80-82 | to the 5’ UTR of neighbor of transcription coactivator (AT5G03500), mRNA at -38 nt |
| 83 | in frame to the 401^st^ aa of RHM1 |
| 84 | in frame to the 5’ UTR of neighbor of ATPC1; enzyme regulator (ATPC1), mRNA at -30 nt (NM_116702) |
| 85 | in frame to the 195^th^ aa of unknown protein (AT1G80210) (NM_106670) |
| 86 | in frame to the 229^th^ aa of DHS2 (3-deoxy-d-arabino-heptulosonate 7-phosphate synthase); 3-deoxy-7-phosphoheptulonate synthase (DHS2) (NM_119505) |
| 87 | in frame to the 28^th^ aa of unknown protein (AT1G49700) (NM_103857) |
| 88 | in frame to the 31^st^ aa of unknown protein (AT3G10250) (NM_111860) |
| 89 | out of frame to the 353^rd^ aa of EMB2750 (embryo defective 2750) (EMB2750) (NM_111518) |
| 90 | in frame to the 3’ UTR of tRNA-splicing endonuclease positive effector-related (AT4G30100) at 4151^st^ nt (NM_119156) |
